# Identification of Risk Factors for Stroke in China: A Meta-Analysis of Prospective Cohort Studies

**DOI:** 10.3389/fneur.2022.847304

**Published:** 2022-03-18

**Authors:** Weizhuang Yuan, Bo Wu, Min Lou, Bo Song, Xiang Han, Feng Sheng, Weihai Xu

**Affiliations:** ^1^Department of Neurology, State Key Laboratory of Complex Severe and Rare Diseases, Peking Union Medical College Hospital, Chinese Academy of Medical Sciences and Peking Union Medical College, Beijing, China; ^2^Department of Neurology, West China Hospital, Sichuan University, Chengdu, China; ^3^Department of Neurology, The Second Affiliated Hospital of Zhejiang University, School of Medicine, Hangzhou, China; ^4^Department of Neurology, The First Affiliated Hospital of Zhengzhou University, Zhengzhou, China; ^5^Department of Neurology, Huashan Hospital, Fudan University, Shanghai, China; ^6^Medical Development, Amgen Biology Technology Consulting (Shanghai) Co., Ltd., Shanghai, China

**Keywords:** stroke, risk factor, primary prevention, Chinese, meta–analysis

## Abstract

This study aimed to identify independent risk factors for first occurrence of stroke in Chinese individuals based on prospective cohort studies. Forty prospective cohort studies assessing 1,984,552 individuals were selected for the final meta-analysis. The identified risk factors for stroke in the Chinese population included old age (RR = 1.86, 95%CI: 1.47–2.36), hypertension (RR = 2.76, 95%CI: 2.26–3.37), cardiovascular disease history (RR = 1.98, 95%CI: 1.06–3.69), chronic kidney disease (RR = 1.65, 95%CI: 1.36–2.01), diabetes mellitus (RR = 1.71, 95%CI: 1.34–2.18), metabolic syndrome (RR = 1.59, 95%CI: 1.33–1.90), hyperglycemia (RR = 1.49, 95% CI: 1.31–1.69), obesity (RR = 1.45, 95%CI: 1.29–1.63), smoking (RR = 1.42, 95% CI: 1.27–1.58), prolonged sleep time (> 7.5 h, RR = 1.44, 95%CI: 1.19–1.75), higher levels of triglyceride (RR = 1.19, 95%CI: 1.07-1.32), C-reactive protein (RR = 1.34, 95%CI: 1.07-1.69). High fruit-rich diet (RR = 0.68, 95%CI: 0.58-0.80) was associated with a lower risk of stroke. The spectrum and power of risk factors varied among different cohort inclusion years. These findings provide a comprehensive tool for the primary prevention of stroke in Chinese individuals.

## Introduction

Stroke is considered the second most common cause of death and disability-adjusted life years (DALYs) among adults aged 50 years or over, according to the Global Burden of Disease Study 2019 (1). There are estimated 7.74 and 4.19 million incident cases of acute first ischemic stroke (IS) and hemorrhagic stroke (HS) worldwide, respectively (2). Studies have indicated that stroke development could be affected by several modifiable risk factors, including elevated lipid profiles, blood pressure (BP), diabetes mellitus (DM), smoking, alcohol, obesity, unhealthy diet, psycho-social stress, and lack of physical activity (PA) (3–5). Via risk factor management including lifestyle intervention (e.g., smoking cessation) and pharmacological intervention (e.g., blood pressure-lowering medication), the age-standardized incidence of stroke has declined by 8.1% globally from 1990 to 2016, and by 20.3% in countries with high Socio-demographic index. In China, stroke is comparatively much less well-controlled, with the age-standardized incidence of stroke increasing by 5.4% from 1990 to 2016 (6). The prevalence of HS is remarkably higher in China compared with other countries (2). A previous systematic review reported HS prevalence in Chinese individuals is two-fold that of Caucasians (7). Therefore, vigorous and efficient primary preventive actions are urgently in need. Unfortunately, the comprehensive profile of risk factors for stroke in the Chinese population still remains undefined up to now.

In the current study, we performed a meta-analysis of prospective cohort studies in Chinese individuals to identify potential risk factors for first occurrence of stroke. Stratified analyses based on types of stroke were also carried out in this study.

## Methods

### Data Sources, Search Strategy, and Selection Criteria

This comprehensive quantitative meta-analysis was carried out and reported, following the Preferred Reporting Items for Systematic Reviews and Meta-Analysis Statement (8). Studies that were designed as prospective cohort trials and reported effect estimates of the risk of first stroke occurrence in Chinese population were included in this study, with no restriction on publication status. On August 22, 2020, we searched the PubMed, EmBase, and Cochrane library databases with the following core search terms: “stroke” AND “risk factors” AND “Chinese.” The details of the search strategy are presented in Supplemental 1. We restricted the publication language to English. To further identify studies which are potentially eligible, a manual search of retrieved articles was conducted in addition.

The initial study selection by reviewing titles and abstracts was conducted by two authors independently, followed by the full-text evaluation based on group discussion involving 3 authors independently. Any disagreement among the authors was settled by the corresponding author after reviewing the original articles. Studies satisfying the following inclusion criteria were included in the meta-analysis: (1) prospective cohort design; (2) assessed risk factors including demographic features, pre-existing diseases, lifestyles, and biochemical exposures reported in ≥ 3 studies; (3) reported original data or adjusted effect estimates (odds ratio [OR], relative risk [RR], or hazard ratio [HR]) with 95% confidence interval (CI); (4) inclusion of healthy Chinese individuals aged ≥18.0 years without stroke history.

### Data Collection and Quality Assessment

Data extraction and quality assessment were carried out by 2 authors. Any disagreement among the authors was settled by an additional author reviewing the original articles. The extracted data were entered in a standardized data collection form. The collected items included name of the first author or the whole study group, publication year, sample size, mean participant age, number of men/women, follow-up duration, reported outcomes, adjusted factors, and effect estimates of the risk of stroke. The Newcastle-Ottawa Scale (NOS), a comprehensive tool and partially validated method for assessing the quality of observational studies (9), was used to assess the quality of included studies. The “star system” of NOS, including selection, comparison and outcome, ranged from 0 to 9. A study with 7 or more stars was considered of high quality.

### Statistical Analysis

The profile of risk factors for first occurrence of stroke in Chinese individuals was examined as binary data. Given the low incidence of stroke, the OR could be regarded as equal to the RR. Moreover, the HR was considered to be approximately equal to RR because the included studies were designed as prospective cohort trials. Then, the pooled RR was calculated by a random-effects model for each parameter (10). Heterogeneity across the included studies for each risk factor was assessed by the *I*^2^ index and *Q* statistic; significant heterogeneity was considered with a *P*-value for *Q* statistic <0.10 (11). Sensitivity analysis was carried out for factors reported in ≥ 10 cohorts by sequentially excluding individual studies (12). Regression analysis was employed to assess the role of cohort inclusion years in the risk of stroke if the factor was reported in ≥ 5 cohorts. Subgroup analysis was also conducted according to types of stroke and cohort inclusion years (inclusion year before 2000 vs. after 2000), while the interaction *P-*value was calculated to assess the differences between subgroups (13). Publication bias for factors reported in ≥ 10 cohorts was also evaluated using funnel plots, and the Egger and Begg tests (14). The *P-*value for all pooled results was two-sided, and *P* < 0.05 was considered statistically significant. The STATA software (version 10.0; Stata Corporation, College Station, TX, USA) was employed for data analysis.

## Results

### Literature Search

**Figure 1:**
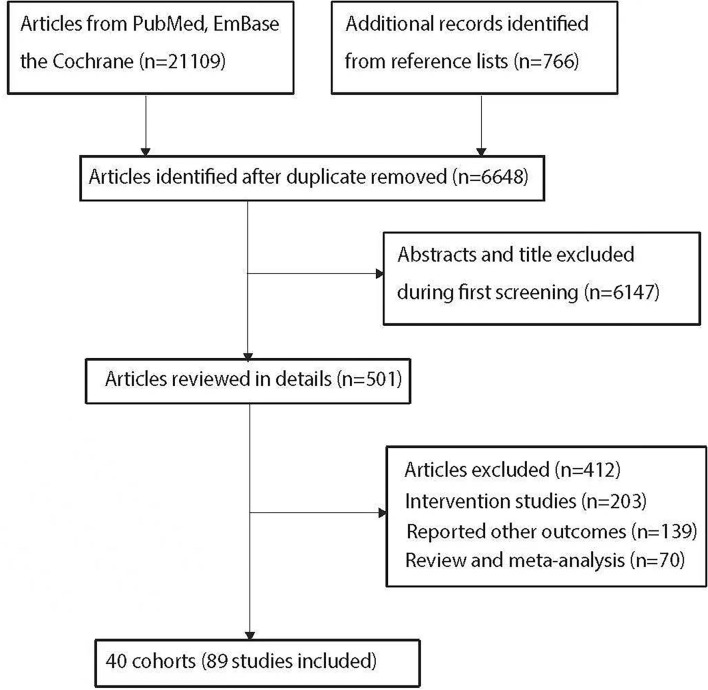
The PRISMA flowchart for literature search and study selection.

The initial electronic search yielded 21,109 articles, and 14,461 were excluded owing to duplicate titles. The remaining 6,648 articles were reviewed by titles and abstracts, and 6,147 were further excluded due to topic irrelevance. A total of 501 studies were retrieved for full-text evaluation, and 40 cohorts reported in 89 studies assessing 1,984,552 individuals were selected for final quantitative meta-analysis. A further manual search throughout the reference lists of included studies yielded no more eligible studies. Details concerning literature search and the study selection process are shown in Figure 1.

### Study Characteristics

Supplemental 2 summarizes the baseline characteristics of included studies and examined populations. The included studies were published in 1996–2019, and each included 421-489,301 individuals. The follow-up duration ranged from 1.0 to 30.0 years. Three of the included cohorts reported unadjusted effect estimates, and the remaining 37 reported adjusted effect estimates. The quality of included studies was assessed by the NOS. Eight studies had 8 stars, 21 had 7 stars, and the remaining 11 had 6 stars.

### Meta-Analysis

**Figure 2:**
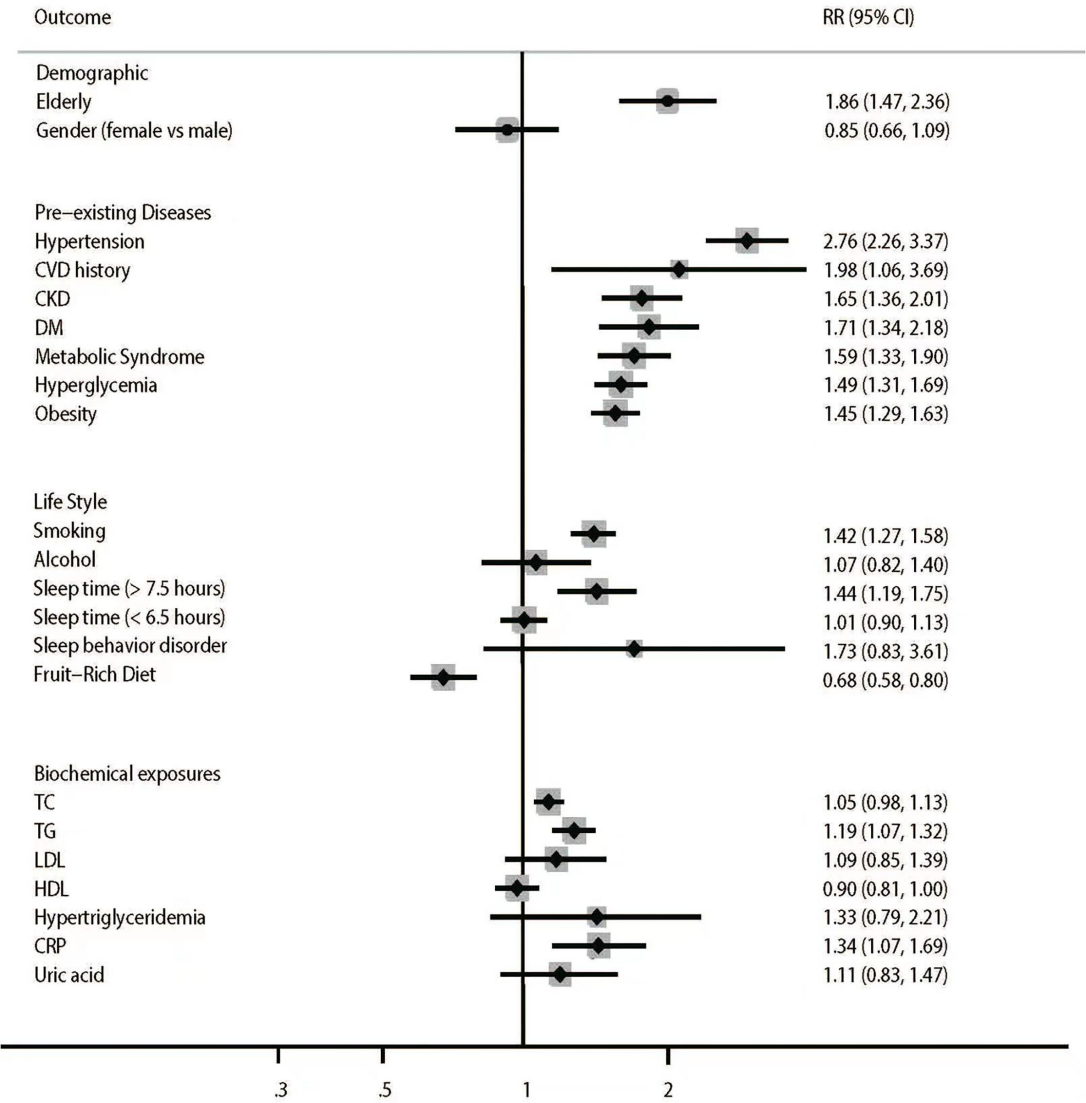
Summary risk factors for stroke incidence.

The pooled results for stroke risk factor profiles are summarized in Figure 2 and Table 1.

**Table 1:** The summary results for risk factors.

**Outcomes**	**Number of cohorts**	**RR and 95%CI**	***P*-value**	**Heterogeneity (%)**	***P*-value for heterogeneity**
**Demographic**					
Elderly	5	1.86 (1.47–2.36)	<0.001	42.0	0.111
Sex (female vs. male)	5	0.85 (0.66–1.09)	0.191	72.5	0.003
**Pre-existing diseases**					
Hypertension	19	2.76 (2.26–3.37)	<0.001	95.8	<0.001
CVD history	4	1.98 (1.06–3.69)	0.033	83.7	<0.001
CKD	6	1.65 (1.36–2.01)	<0.001	47.7	0.046
DM	7	1.71 (1.34–2.18)	<0.001	63.3	0.012
Metabolic Syndrome	6	1.59 (1.33–1.90)	<0.001	22.7	0.249
Hyperglycemia	7	1.49 (1.31–1.69)	<0.001	0.0	0.531
Obesity	15	1.45 (1.29–1.63)	<0.001	74.3	<0.001
**Life style**					
Smoking	15	1.42 (1.27–1.58)	<0.001	78.0	<0.001
Alcohol	5	1.07 (0.82–1.40)	0.626	77.8	0.001
Sleep time (> 7.5 h)	4	1.44 (1.19–1.75)	<0.001	76.7	<0.001
Sleep time (<6.5 h)	4	1.01 (0.90–1.13)	0.917	44.0	0.098
Sleep behavior disorder	3	1.73 (0.83–3.61)	0.146	84.4	0.002
Fruit-Rich Diet	3	0.68 (0.58–0.80)	<0.001	69.9	0.036
**Biochemical exposures**					
TC	4	1.05 (0.98–1.13)	0.151	35.4	0.158
TG	6	1.19 (1.07–1.32)	0.001	67.0	0.002
LDL	3	1.09 (0.85–1.39)	0.502	75.6	0.003
HDL	6	0.90 (0.81–1.00)	0.062	77.8	<0.001
Hypertriglyceridemia	3	1.33 (0.79–2.21)	0.280	78.3	0.010
CRP	3	1.34 (1.07-1.69)	0.012	25.3	0.262
Uric acid	3	1.11 (0.83–1.47)	0.490	67.7	0.045

The summary RRs indicated elderly (RR = 1.86, 95%CI: 1.47–2.36, *P* < 0.001) was associated with increased risk of stroke, whereas no sex difference was found regarding the risk of stroke (RR = 0.85, 95%CI: 0.66–1.09, *P* = 0.191). Pre-existing hypertension (RR = 2.76, 95%CI: 2.26–3.37; *P* < 0.001), cardiovascular disease (CVD) history (RR = 1.98, 95%CI: 1.06–3.69; *P* = 0.033), chronic kidney disease (CKD) (RR = 1.65, 95%CI: 1.36–2.01, *P* < 0.001), diabetes mellitus (DM) (RR = 1.71, 95%CI: 1.34–2.18, *P* < 0.001), metabolic syndrome (RR = 1.59, 95% CI: 1.33–1.90, *P* < 0.001), hyperglycemia (RR = 1.49, 95% CI: 1.31–1.69, *P* < 0.001) and obesity (RR = 1.45, 95%CI: 1.29–1.63, *P* < 0.001) were associated with increased risk of stroke. Unhealthy lifestyles, including smoking (RR = 1.42, 95%CI: 1.27–1.58, *P* < 0.001) and prolonged sleep time (>7.5 h) (RR = 1.44, 95%CI: 1.19–1.75, *P* < 0.001), increased the risk of stroke. Meanwhile, alcohol intake, short sleep time (<6.5 h), and sleep behavior disorder were not associated with the risk of stroke. Besides, higher fruit-rich diet intake led to a reduced risk of stroke (RR = 0.68, 95%CI: 0.58–0.80, *P* < 0.001). In addition, higher levels of triglycerides (TG) (RR = 1.19, 95%CI: 1.07–1.32, *P* = 0.001) and C-reactive protein (CRP) (RR = 1.34, 95%CI: 1.07–1.69, *P* = 0.012) were associated with increased risk of stroke, whereas TC, LDL, HDL, hypertriglyceridemia, and uric acid were not associated with the risk of stroke in the Chinese population. Significant heterogeneity was observed for sex (*I*^2^= 72.5%; *P* = 0.003), hypertension (*I*^2^= 95.8%; *P* < 0.001), CVD history (*I*^2^= 83.7%; *P* < 0.001), CKD (*I*^2^= 47.7%; *P* = 0.046), DM (*I*^2^= 63.3%; *P* = 0.012), obesity (*I*^2^= 74.3%; *P* < 0.001), smoking (*I*^2^= 78.0%; *P* < 0.001), alcohol intake (*I*^2^= 77.8%; *P* = 0.001), prolonged sleep time (>7.5 h) (*I*^2^= 76.7%; *P* < 0.001), short sleep time (<6.5 h) (*I*^2^= 44.0%; *P* = 0.098), sleep behavior disorder (*I*^2^= 84.4%; *P* = 0.002), fruit-rich diet (*I*^2^= 69.9%; *P* = 0.036), TG (*I*^2^= 67.0%; *P* = 0.002), LDL (*I*^2^= 75.6%; *P* = 0.003), HDL (*I*^2^= 77.8%; *P* < 0.001), hypertriglyceridemia (*I*^2^= 78.3%; *P* = 0.010), and uric acid (*I*^2^= 67.7%; *P* = 0.045). No other significant heterogeneity across the included studies was observed. The associations of hypertension, obesity, and smoking with the risk of stroke in Chinese individuals were reported in ≥ 10 cohorts, and sensitivity analysis suggested that the pooled conclusions were robust irrespective of excluded studies (Supplemental 3).

### Subgroup Analysis

Subgroup analysis (Table 2) suggested that hypertension, CVD history, CKD, hyperglycemia and obesity were associated with the risk of both IS and HS. The RRs of hypertension and sleep behavior disorder were high in HS, while that of obesity was high in IS. Subgroup analysis also showed that DM, metabolic syndrome, smoking, prolonged sleep time (>7.5 h), and elevated TC, TG, and CRP were only associated with the risk of IS. Alcohol intake was only associated with a reduced risk of IS, while not significantly affecting the risk of HS. A fruit-rich diet could protect the body against both IS and HS risk, and the protective effect was stronger on HS than on IS.

**Table 2:** Subgroup analysis of stroke subtypes.

**Factor**	**Subgroup**	**Number of studies**	**RR and 95%CI**	***P*-value**	**Heterogeneity**	***P*-value between subgroups**
Sex (female vs. male)	IS	4	0.93 (0.57–1.51)	0.769	83.8 (<0.001)	0.376
	HS	2	0.70 (0.53–0.91)	0.008	53.4 (0.143)	
Hypertension	IS	12	2.36 (1.93–2.89)	<0.001	72.5 (<0.001)	<0.001
	HS	6	3.67 (2.28–5.92)	<0.001	89.3 (<0.001)	
CVD history	IS	2	2.21 (1.25–3.91)	0.006	5.4 (0.304)	0.422
	HS	1	2.82 (2.07–3.85)	<0.001	-	
CKD	IS	9	1.57 (1.24–1.99)	<0.001	50.4 (0.041)	0.106
	HS	2	2.25 (1.52–3.35)	<0.001	0.0 (0.751)	
DM	IS	2	1.92 (1.51–2.45)	<0.001	35.7 (0.212)	0.005
	HS	2	1.27 (0.89–1.81)	0.180	64.2 (0.095)	
Metabolic Syndrome	IS	4	1.72 (1.24–2.38)	0.001	61.0 (0.053)	0.361
	HS	2	1.36 (0.99–1.88)	0.057	0.0 (0.650)	
Hyperglycemia	IS	5	1.45 (1.13–1.85)	0.003	30.2 (0.220)	0.904
	HS	4	1.45 (1.20–1.77)	<0.001	0.0 (0.604)	
Obesity	IS	13	1.73 (1.51–1.98)	<0.001	69.6 (<0.001)	<0.001
	HS	11	1.31 (1.08–1.58)	0.005	66.0 (0.001)	
Smoking	IS	10	1.63 (1.46–1.83)	<0.001	0.0 (0.500)	0.060
	HS	6	1.18 (0.85–1.63)	0.318	67.6 (0.009)	
Alcohol	IS	1	0.76 (0.63–0.92)	0.004	-	0.326
	HS	1	0.92 (0.66–1.28)	0.622	-	
Sleep time (> 7.5 hours)	IS	3	1.32 (1.02–1.70)	0.035	76.8 (0.013)	0.202
	HS	3	1.15 (0.87–1.52)	0.329	36.5 (0.207)	
Sleep time (<6.5 hours)	IS	3	1.07 (0.84–1.37)	0.563	82.6 (0.003)	0.575
	HS	3	0.97 (0.81–1.17)	0.776	0.0 (0.734)	
Sleep behavior disorder	IS	1	1.93 (1.07–3.47)	0.028	-	0.048
	HS	1	6.61 (2.27–19.26)	0.001	-	
Fruit-rich diet	IS	1	0.75 (0.72–0.79)	<0.001	-	0.034
	HS	1	0.64 (0.56–0.74)	<0.001	-	
TC	IS	4	1.13 (1.04–1.23)	0.005	30.7 (0.217)	0.001
	HS	3	0.93 (0.84–1.02)	0.119	2.9 (0.378)	
TG	IS	4	1.20 (1.05–1.38)	0.010	67.6 (0.015)	0.525
	HS	3	1.13 (0.95–1.36)	0.164	64.0 (0.040)	
LDL	IS	2	1.42 (0.84–2.38)	0.190	89.0 (0.003)	0.019
	HS	3	0.92 (0.77–1.10)	0.339	0.0 (0.418)	
HDL	IS	4	0.95 (0.90–1.01)	0.108	0.0 (0.702)	0.001
	HS	3	0.89 (0.72–1.10)	0.285	87.4 (<0.001)	
Hypertriglyceridemia	IS	1	1.36 (0.98–1.89)	0.066	-	0.371
	HS	2	1.21 (0.40–3.65)	0.734	88.1 (0.004)	
CRP	IS	2	1.31 (1.10–1.57)	0.003	0.0 (0.419)	0.227
	HS	2	1.05 (0.77–1.44)	0.738	0.0 (0.449)	
Uric acid	IS	2	1.13 (0.80–1.60)	0.475	70.2 (0.067)	0.495
	HS	2	0.93 (0.54–1.61)	0.791	64.8 (0.092)	

Stratified analyses based on cohort inclusion years were also conducted, and cohort inclusion years could bias the associations of sex (female vs. male), hypertension, DM, obesity, smoking, and elevated TG and HDL with the risk of stroke (Table 3). In pooled cohorts initially recruited before 2000, hypertension, CKD, DM, hyperglycemia, obesity, and smoking were associated with increased risk of stroke. Furthermore, in pooled cohorts initially recruited in 2000 or afterwards, hypertension, DM, metabolic syndrome, hyperglycemia, smoking, and elevated TG were associated with increased risk of stroke. Finally, the female sex and elevated HDL decreased the risk of stroke when pooled cohorts were initially recruited in or after 2000.

**Table 3:** Subgroup analysis of stroke incidence based on inclusion period.

**Factor**	**Inclusion period**	**RR and 95%CI**	***P-*value**	**Heterogeneity (%)**	***P*-value for heterogeneity**	***P*-value between subgroups**
Sex (female vs. male)	2000 or after	0.64 (0.56–0.73)	<0.001	0.0	0.650	<0.001
	Before 2000	1.06 (0.82–1.37)	0.658	21.6	0.281	
Hypertension	2000 or after	2.33 (1.82–2.96)	<0.001	71.6	<0.001	<0.001
	Before 2000	3.04 (2.32–3.98)	<0.001	97.2	<0.001	
CKD	2000 or after	1.36 (0.77–2.40)	0.282	77.2	0.004	0.312
	Before 2000	1.79 (1.51–2.12)	<0.001	0.0	0.695	
DM	2000 or after	1.44 (1.20–1.73)	<0.001	27.6	0.246	0.001
	Before 2000	2.53 (1.91–3.34)	<0.001	0.0	0.620	
Metabolic Syndrome	2000 or after	1.62 (1.37–1.92)	<0.001	0.0	0.749	0.549
	Before 2000	2.05 (0.88–4.76)	0.096	70.4	0.034	
Hyperglycemia	2000 or after	1.48 (1.27–1.71)	<0.001	1.6	0.406	0.858
	Before 2000	1.52 (1.19–1.93)	0.001	0.0	0.404	
Obesity	2000 or after	1.26 (0.97–1.63)	0.077	85.1	<0.001	0.037
	Before 2000	1.51 (1.30–1.76)	<0.001	54.0	0.026	
Smoker	2000 or after	1.46 (1.27–1.67)	<0.001	0.0	0.321	0.014
	Before 2000	1.40 (1.24–1.58)	<0.001	80.0	<0.001	
TG	2000 or after	1.50 (1.28–1.77)	<0.001	0.0	0.959	<0.001
	Before 2000	1.09 (1.00–1.18)	0.052	47.8	0.088	
HDL	2000 or after	0.82 (0.69–0.97)	0.022	52.3	0.123	<0.001
	Before 2000	0.96 (0.91–1.01)	0.146	0.0	0.423	

Next, the potential role of cohort inclusion years in pooled effect estimates was investigated for factors reported in ≥5 cohorts (Supplemental 4). The results showed that the cumulative RRs of hypertension (*P* = 0.020) and obesity (*P* = 0.046) in HS decreased with increasing inclusion years. However, no association was observed between cohort inclusion years and the effects of alcohol, CKD, DM, sex, hyperglycemia, hypertension, metabolic syndrome, obesity, and smoking on the risk of all types of stroke. Furthermore, the associations of CKD, hypertension, obesity, and smoking with the risk of IS were not affected by cohort inclusion years. Finally, cohort inclusion years did not affect the association between smoking and the risk of HS.

### Publication Bias

Publication bias was evaluated for hypertension, obesity, and smoking (Supplemental 5). There was no significant publication bias for hypertension (*P*_Egger_ = 0.468; *P*_Begg_ = 0.113). Although the Begg's test indicated no significant publication bias for obesity (*P* = 0.944) and smoking (*P* = 0.150), the Egger's test suggested potential significant publication bias for obesity (*P* = 0.007) and smoking (*P* = 0.051). After adjustment by the trim and fill method, the pooled conclusions were not significantly changed (Supplemental 5).

## Discussion

### Principal Findings

This comprehensive quantitative meta-analysis assessed 1,984,552 individuals in 40 prospective cohorts across wide individual characteristics. The results suggested the demographic characteristic of old age; pre-existing diseases such as hypertension, CVD history, CKD, DM, metabolic syndrome, hyperglycemia, and obesity; unhealthy lifestyles, including smoking and prolonged sleep time (>7.5 h); and the biochemical parameters of high TG and CRP levels were all associated with increased risk of stroke in Chinese individuals. Only high fruit-rich diet intake could decrease the risk of stroke. Subgroup analysis suggested the RRs of hypertension and sleep behavior disorder were high in HS, while that of obesity was high in IS. The protective effect of fruit-rich diet was stronger in HS compared with IS. It was also found that the cumulative RRs of hypertension and obesity in HS decreased with increasing inclusion years.

### Comparison With Previous Studies

A previous meta-analysis by Wang et al. including only 15 cohorts and 178 case-control studies found hypertension, DM, CVD history, family history of stroke, hyperlipidemia, overweight, and smoking were correlated with high risk of stroke, while PA could reduce the risk of stroke (15). The uncontrolled selection and confounder biases might affect the overall reliability of pooled effect estimates, with both prospective and retrospective observational studies included. In addition, the results regarding single variables in previous meta-analyses are presented in Supplemental 6 (4, 16–32). Although most pooled conclusions in Chinese individuals were consistent with previous meta-analyses, several results should be highlighted. (1) The present study found obesity increased the risk of stroke irrespective of IS or HS, whereas a previous meta-analysis found overweight and obesity increased the risk of stroke with a J-shaped dose-response relationship (4). The potential reason for this discrepancy could be the variable cutoff values for obesity and overweight in individual studies. (2) As shown above, alcohol intake was associated with reduced risk of IS. In comparison, a previous meta-analysis found low-to-moderate alcohol intake was associated with lower risk of IS, whereas heavy alcohol intake increased the risk of both IS and HS (33). A dose-response curve was not generated in this study, because restricted cubic splines with 3 knots at the fixed percentiles of 10, 50, and 90% of distribution were reported in few studies (34). (3) Prolonged sleep time was associated with increased risk of stroke, whereas a previous meta-analysis found both prolonged and short sleep times were correlated with high risk of stroke (21). The potential reason for this difference could be attributed to the small number of included cohorts, and the insufficient statistical power that fails to detect tiny difference.

### Implications for Clinicians and Policy Makers

Stratified analyses suggested risk factors for HS and IS differed. Cohort inclusion years could affect the impacts of these risk factors. Several issues should be highlighted based on these findings. (1) As shown above, hypertension was the main risk factor for HS and exerted more effects on HS than on IS. We also found the risk of hypertension decreased for HS while remaining unchanged for IS, and the accumulative RR for hypertension decreased with increasing inclusion years. This may be explained by the current status of hypertension control in China. Hypertension control rates are increasing, coinciding with a rising hypertension prevalence across the nation. According to six national epidemiological investigations, the awareness (26.3 vs. 51.6%), treatment (12.1 vs. 45.8%), and control (2.8% vs. 16.8%) rates for hypertension increased from 1991 to 2015, while hypertension prevalence increased from 5.1% (in 1959) to 7.7% (in 1980), 13.6% (in 1991), 18.8% (in 2002), 25.2% (in 2012), and 27.9% (in 2015) (35). We can therefore infer that hypertension control in China is active but not robust enough. Strategies for hypertension management should be strengthened, and the risk of IS would decrease. (2) Obesity showed a tighter association with the risk of IS compared with HS. Actually, obesity is associated with various ischemic risk factors for stroke, including hypertension, dyslipidemia, DM, obstructive sleep apnea syndrome, and atrial fibrillation. Controlling obesity could decrease the risk of stroke directly and help better manage other risk factors. Similarly, the decreasing adverse impact of obesity on the risk of HS could be partly explained by hypertension, dyslipidemia and DM control strategies implemented in China, while obesity prevalence among Chinese adults has increased substantially with the development of economy (36). These data suggest there is an urgent need to reverse the trend toward obesity. (3) As shown above, fruit-rich diet was a strong protective factor for stroke in China, which remains consistent with previous studies (37, 38). The mechanisms underlying protection from fruits vary by fruit type, including the modulation of molecular events and signaling pathways associated with correcting endothelial dysfunction, reducing disorders in lipid metabolism, anti-hypertension, suppressing platelet function, alleviating I/R injury, inhibiting thrombosis, reducing oxidative stress, and inhibiting inflammatory responses (39). The reasons why fruit-rich diet was associated with lower risk of HS compared with IS in this study deserve further investigation; but fruit-rich diet should be encouraged in our future primary stroke prevention strategy anyway. To meet the challenge of stroke as a major cause of mortality, and long-term physical and cognitive impairment, China launched a nationwide project to promote stroke prevention and control (40). The program has led to a significant improvement in the care for stroke patients. The results of our study suggested similar spectrum of risk factors and preventive strategy in Chinese individuals for stroke when compared with their foreign counterparts, which indicated that clinicians in China can also handle individuals at higher risk of stroke referring to guidelines and evidence from other countries.

### Unanswered Questions and Future Research

There has been an increasing interest in novel nontraditional risk factors, some of which are considered to be specifically important in the Chinese population but are not included here due to the limited number of studies taken in. Taking air pollution as an example, the relation between exposure to fine particles and stroke has been reported previously (41). It was shown that air pollution accounts for almost a third of stroke-related disability-adjusted life years, especially in China. Therefore, further prospective studies assessing Chinese individuals are needed, and reducing exposure to air pollution should be one of the top priorities in order to reduce stroke burden. Besides, sleep behavior disorder was a considerable risk factor. However, whether the intervention of sleep behavior disorder could decrease the risk of stroke remains unknown and needs further research.

### Strengths and Limitations of Study

The strengths of this comprehensive, quantitative meta-analysis should be highlighted: (1) this study was based on prospective cohort trials, and selection and recall biases could eliminate the concerns of retrospective observational studies; (2) the analysis was based on a large sample size, and the present conclusions were robust than that of any individual study; (3) comprehensive risk factor profiles for the incidence of stroke were provided; and (4) the analysis was stratified by stroke type and cohort inclusion years, which helped explore the potential differences between IS and HS and avoid selection bias.

However, several limitations of this meta-analysis should be acknowledged: (1) the adjusted models were different among the included studies, which might play an important role in the progression of stroke; (2) for certain identified factors, only small numbers of included studies were available, and as a result, the statistical power might not be enough to detect potential associations; (3) this study was based on published articles, and publication bias was therefore inevitable; and (4) the current analysis was based on study-level findings, and individual data were not available, which prevented a more detailed analysis.

## Conclusion

We provided a comprehensive updated set of risk factors for stroke in Chinese individuals, which would inspire the design of interventional studies and support future development in guideline or policy for stroke prevention.

## Data Availability Statement

The raw data supporting the conclusions of this article will be made available by the authors, without undue reservation.

## Author Contributions

WX was the study guarantor and designed the review protocol. FS developed the search strategy. WY and FS selected the studies and extracted the data. WX and WY analyzed the data and drafted the manuscript. BW, ML, BS, XH, and FS revised the manuscript for important intellectual content. All authors approved the final version of the manuscript. All authors had access to all the data in the study and take responsibility for the integrity of these data and the accuracy of data analysis.

## Funding

The funder had the following involvement: data collection. The funder was not involved in the study design, analysis, interpretation of data, the writing of this article or the decision to submit it for publication.

## Conflict of Interest

FS was employed by Amgen. This study received funding from Amgen. The funder was involved with data collection. The remaining authors declare that the research was conducted in the absence of any commercial or financial relationships that could be construed as a potential conflict of interest. The reviewer W-JT declared a shared affiliation, with no collaboration, with several of the authors WY and WX to the handling editor at the time of the review.

## Publisher's Note

All claims expressed in this article are solely those of the authors and do not necessarily represent those of their affiliated organizations, or those of the publisher, the editors and the reviewers. Any product that may be evaluated in this article, or claim that may be made by its manufacturer, is not guaranteed or endorsed by the publisher.

## Abbreviations

BP: blood pressure
CI: confidence interval
CKD: chronic kidney disease
CRP: C-reactive protein
CVD: cardiovascular disease
DM: diabetes mellitus
HR: hazard ratio
HS: hemorrhagic stroke
IS: ischemic stroke
NOS: Newcastle-Ottawa Scale
OR: odds ratio
PA: physical activity
RR: relative risk
TG: triglycerides.
