# Supplementary material for: Identification of Risk Factors for Stroke in China: A Meta-Analysis of Prospective Cohort Studies
Source: Front Neurol. 2022 Mar 18;13:847304. doi: 10.3389/fneur.2022.847304 (PMC8972128; doi:10.3389/fneur.2022.847304)
Supplement: Supplementary file 1 [file Table_1.DOCX]

**Search strategy in PubMed and the Cochrane library:**

| Search | Query |
| --- | --- |
| #8 | Search (English[Language]) AND (((((((((((((((brain[Title/Abstract] OR cerebr*[Title/Abstract] OR cerebell*[Title/Abstract] OR vertebrobasil*[Title/Abstract] OR hemispher*[Title/Abstract] OR intracran*[Title/Abstract] OR intracerebral[Title/Abstract] OR infratentorial[Title/Abstract] OR supratentorial[Title/Abstract] OR middle cerebr*[Title/Abstract] OR mca*[Title/Abstract] OR anterior circulation[Title/Abstract] OR basilar artery[Title/Abstract] OR vertebral artery[Title/Abstract])) AND (stroke*[Title/Abstract] OR ischemi*[Title/Abstract] OR infarct*[Title/Abstract] OR thrombo*[Title/Abstract] OR emboli*[Title/Abstract] OR occlus*[Title/Abstract] OR hypoxi*[Title/Abstract]))))))) OR (((((((stroke*[Title/Abstract] OR apoplex*[Title/Abstract] OR cerebral vasc*[Title/Abstract] OR cerebrovasc*[Title/Abstract] OR cva[Title/Abstract] OR attack*[Title/Abstract])) AND ischemi*[Title/Abstract])))))) OR (("brain ischemia"[mh:noexp] or "brain infarction"[mesh] or "carotid artery diseases"[mh:noexp] or "carotid artery thrombosis"[mh:noexp] or "carotid artery, internal, dissection"[mh:noexp] or "intracranial arterial diseases"[mh:noexp] or "cerebral arterial diseases"[mh:noexp] or " infarction, anterior cerebral artery"[mh:noexp] or "infarction, middle cerebral artery"[mh:noexp] or "infarction, posterior cerebral artery"[mh:noexp] or "intracranial embolism and thrombosis"[mesh] or stroke[mesh] or "basal ganglia cerebrovascular disease"[mh:noexp] or "vertebral artery dissection"[mh:noexp]))))) AND ((((((China[Mesh] OR Taiwan[Mesh] OR Hong Kong[Mesh] OR Macau[Mesh] OR china[Affiliation] OR chinese[Affiliation] OR Taiwan[Affiliation] OR Hong Kong[Affiliation] OR Macau [Affiliation] OR china[Title/Abstract] OR chinese[Title/Abstract] OR Taiwan[Title/Abstract] OR Hong Kong[Title/Abstract] OR Macau[Title/Abstract]))))))) AND ((((registr*[Title/Abstract] OR Registries[mesh]))) OR ((((cohort*[Title/Abstract] OR Follow-Up Stud*[Title/Abstract] OR Follow-Up*[Title/Abstract] OR Longitudinal Stud*[Title/Abstract] OR Prospective*[Title/Abstract] OR Prospective Stud*[Title/Abstract] OR Concurrent Stud*[Title/Abstract] OR Incidence Stud*[Title/Abstract] OR "Cohort Studies"[Mesh])))))) |
| #7 | Search English[Language] |
| #6 | Search ((((((((((((((brain[Title/Abstract] OR cerebr*[Title/Abstract] OR cerebell*[Title/Abstract] OR vertebrobasil*[Title/Abstract] OR hemispher*[Title/Abstract] OR intracran*[Title/Abstract] OR intracerebral[Title/Abstract] OR infratentorial[Title/Abstract] OR supratentorial[Title/Abstract] OR middle cerebr*[Title/Abstract] OR mca*[Title/Abstract] OR anterior circulation[Title/Abstract] OR basilar artery[Title/Abstract] OR vertebral artery[Title/Abstract])) AND (stroke*[Title/Abstract] OR ischemi*[Title/Abstract] OR infarct*[Title/Abstract] OR thrombo*[Title/Abstract] OR emboli*[Title/Abstract] OR occlus*[Title/Abstract] OR hypoxi*[Title/Abstract]))))))) OR (((((((stroke*[Title/Abstract] OR apoplex*[Title/Abstract] OR cerebral vasc*[Title/Abstract] OR cerebrovasc*[Title/Abstract] OR cva[Title/Abstract] OR attack*[Title/Abstract])) AND ischemi*[Title/Abstract])))))) OR (("brain ischemia"[mh:noexp] or "brain infarction"[mesh] or "carotid artery diseases"[mh:noexp] or "carotid artery thrombosis"[mh:noexp] or "carotid artery, internal, dissection"[mh:noexp] or "intracranial arterial diseases"[mh:noexp] or "cerebral arterial diseases"[mh:noexp] or " infarction, anterior cerebral artery"[mh:noexp] or "infarction, middle cerebral artery"[mh:noexp] or "infarction, posterior cerebral artery"[mh:noexp] or "intracranial embolism and thrombosis"[mesh] or stroke[mesh] or "basal ganglia cerebrovascular disease"[mh:noexp] or "vertebral artery dissection"[mh:noexp]))))) AND ((((((China[Mesh] OR Taiwan[Mesh] OR Hong Kong[Mesh] OR Macau[Mesh] OR china[Affiliation] OR chinese[Affiliation] OR Taiwan[Affiliation] OR Hong Kong[Affiliation] OR Macau[Affiliation] OR china[Title/Abstract] OR chinese[Title/Abstract] OR Taiwan[Title/Abstract] OR Hong Kong[Title/Abstract] OR Macau[Title/Abstract]))))))) AND ((((registr*[Title/Abstract] OR Registries[mesh]))) OR ((((cohort*[Title/Abstract] OR Follow-Up Stud*[Title/Abstract] OR Follow-Up*[Title/Abstract] OR Longitudinal Stud*[Title/Abstract] OR Prospective*[Title/Abstract] OR Prospective Stud*[Title/Abstract] OR Concurrent Stud*[Title/Abstract] OR Incidence Stud*[Title/Abstract] OR "Cohort Studies"[Mesh]))))) |
| #5 | Search (((registr*[Title/Abstract] OR Registries[mesh]))) OR ((((cohort*[Title/Abstract] OR Follow-Up Stud*[Title/Abstract] OR Follow-Up*[Title/Abstract] OR Longitudinal Stud*[Title/Abstract] OR Prospective*[Title/Abstract] OR Prospective Stud*[Title/Abstract] OR Concurrent Stud*[Title/Abstract] OR Incidence Stud*[Title/Abstract] OR "Cohort Studies"[Mesh])))) |
| #4 | Search (registr*[Title/Abstract] OR Registries[mesh]) |
| #3 | Search (((cohort*[Title/Abstract] OR Follow-Up Stud*[Title/Abstract] OR Follow-Up*[Title/Abstract] OR Longitudinal Stud*[Title/Abstract] OR Prospective*[Title/Abstract] OR Prospective Stud*[Title/Abstract] OR Concurrent Stud*[Title/Abstract] OR Incidence Stud*[Title/Abstract] OR "Cohort Studies"[Mesh]))) |
| #2 | Search (((((China[Mesh] OR Taiwan[Mesh] OR Hong Kong[Mesh] OR Macau[Mesh] OR china[Affiliation] OR chinese[Affiliation] OR Taiwan[Affiliation] OR Hong Kong[Affiliation] OR Macau[Affiliation] OR china[Title/Abstract] OR chinese[Title/Abstract] OR Taiwan[Title/Abstract] OR Hong Kong[Title/Abstract] OR Macau[Title/Abstract]))))) |
| #1 | Search (((((((((((brain[Title/Abstract] OR cerebr*[Title/Abstract] OR cerebell*[Title/Abstract] OR vertebrobasil*[Title/Abstract] OR hemispher*[Title/Abstract] OR intracran*[Title/Abstract] OR intracerebral[Title/Abstract] OR infratentorial[Title/Abstract] OR supratentorial[Title/Abstract] OR middle cerebr*[Title/Abstract] OR mca*[Title/Abstract] OR anterior circulation[Title/Abstract] OR basilar artery[Title/Abstract] OR vertebral artery[Title/Abstract])) AND (stroke*[Title/Abstract] OR ischemi*[Title/Abstract] OR infarct*[Title/Abstract] OR thrombo*[Title/Abstract] OR emboli*[Title/Abstract] OR occlus*[Title/Abstract] OR hypoxi*[Title/Abstract]))))))) OR (((((((stroke*[Title/Abstract] OR apoplex*[Title/Abstract] OR cerebral vasc*[Title/Abstract] OR cerebrovasc*[Title/Abstract] OR cva[Title/Abstract] OR attack*[Title/Abstract])) AND ischemi*[Title/Abstract])))))) OR (("brain ischemia"[mh:noexp] or "brain infarction"[mesh] or "carotid artery diseases"[mh:noexp] or "carotid artery thrombosis"[mh:noexp] or "carotid artery, internal, dissection"[mh:noexp] or "intracranial arterial diseases"[mh:noexp] or "cerebral arterial diseases"[mh:noexp] or " infarction, anterior cerebral artery"[mh:noexp] or "infarction, middle cerebral artery"[mh:noexp] or "infarction, posterior cerebral artery"[mh:noexp] or "intracranial embolism and thrombosis"[mesh] or stroke[mesh] or "basal ganglia cerebrovascular disease"[mh:noexp] or "vertebral artery dissection"[mh:noexp]))) |

**Search strategy in EmBase:**

1. brain ischemia/ or exp brain infarction/ or carotid artery diseases/ or carotid artery thrombosis/ or carotid artery, internal, dissection/ or intracranial arterial diseases/ or cerebral arterial diseases/ or infarction, anterior cerebral artery/ or infarction, middle cerebral artery/ or infarction, posterior cerebral artery/ or exp "intracranial embolism and thrombosis"/ or exp stroke/ or basal ganglia cerebrovascular disease/ or vertebral artery dissection/
2. (isch?emi$ adj6 (stroke$ or apoplex$ or cerebral vasc$ or cerebrovasc$ or cva or attack$)).tw.
3. ((brain or cerebr$ or cerebell$ or vertebrobasil$ or hemispher$ or intracran$ or intracerebral or infratentorial or supratentorial or middle cerebr$ or mca$ or anterior circulation or basilar artery or vertebral artery) adj5 (stroke$ or isch?emi$ or infarct$ or thrombo$ or emboli$ or occlus$ or hypoxi$)).tw.
4. Or/1-3
5. exp China/
6. exp Taiwan/
7. exp Hong Kong/
8. exp Macao/
9. (china or chinese or taiwan or "hong kong" or macau or macao).tw,kw,in. (2084202)
10. or/4-9
11. (Cohort* or "follow-up stud*" or "follow-up" or "longitudinal stud*" or prospective* or "prospective stud*" or "concurrent stud*" or "incidence stud*" or "cohort studies" or regist*).tw,kw.
12. exp Cohort analysis/
13. exp Follow up/
14. exp Longitudinal study/
15. exp Prospective study/
16. exp Register/
17. or/11-16
18. or 4 and 10 and 117
19. limit 18 to english language
