# Supplementary material for: Identification of Risk Factors for Stroke in China: A Meta-Analysis of Prospective Cohort Studies
Source: Front Neurol. 2022 Mar 18;13:847304. doi: 10.3389/fneur.2022.847304 (PMC8972128; doi:10.3389/fneur.2022.847304)
Supplement: Supplementary file 2 [file Table_2.DOCX]

Table 1. Baseline characteristics of the included studies

| Study | Publication year | Time  period | Sample size | Mean age (years) | Number of men/women | Follow-up duration | Reported outcomes | Adjusted factors | Study quality |
| --- | --- | --- | --- | --- | --- | --- | --- | --- | --- |
| Yuan [1] | 1996 | 1986.1-1993.9 | 18244 | 45.0-64.0 | 18244/0 | 5.4 years | All stroke | Crude | 7 |
| Hsu [2] | 2004 | 1989-1996 | 4049 | >60.0 | 2311/1738 | 7.0 years | All stroke | Crude | 6 |
| Zhang[3] | 2004 | 1974-1993 | 5092 | 18.0-74.0 | 5092/0 | 13.5 years | IS, HS, and all stroke | Age, blood pressure, TC, BMI, and smoking | 7 |
| CVD-FACTS[4,5] | 2006 | 1991-2001 | 3453 | 46.4 | 1499/1954 | 10.4 years | IS | Age, age squared, residential township, smoking, alcohol intake, PA, parental history of stroke, and education level. | 7 |
| Fang[6] | 2006 | 1987-1997 | 26587 | 51.5 | 12560/14027 | 8.8 years | IS, HS, and all stroke | Age, BMI, smoking, alcohol drinking, existing any kind of heart disease and DM, community lived, and antihypertensive treatment | 7 |
| CNHS [7-10] | 2007 | 1991-2000 | 64338 | 55.7 | 64338/0 | 7.7 years | IS, HS, and all stroke | Age, BMI, PA, urban or rural residence, northern or southern China, cigarette smoking, DM, and education | 7 |
| Chen[11] | 2007 | 1989-2003 | 2453 | 71.4 | 1247/1206 | 14.0 years | All stroke | Age, gender, SBP, DBP, BMI, disability, smoking, alcohol drinking, history of CHD, DM and stroke | 6 |
| Hong Kong Diabetes Registry [12,13] | 2008 | 1995-2005 | 6445 | 56.5 | 2928/3517 | 5.4 years | IS | Age, sex, smoking status, hypertension, BMI, HDL, duration of DM, eGFR, and use of drugs at baseline | 7 |
| Zhou [14] | 2008 | 1990-2001 | 211946 | 40.0-79.0 | 211946 | 10.0 years | IS and HS | Age, area, smoking, and alcohol drinking | 7 |
| MJ Health Screening Cohort [15] | 2009 | 1994-2003 | 90393 | 51.5 | 41879/48514 | 8.2 years | IS | Age, sex, BMI, cholesterol, triglycerides, DM, hypertension, heavy cigarette smoking, and frequent alcohol consumption | 7 |
| The CCCC study [16-21] | 2009 | 1990-2005 | 3513 | 61.8 | 1703/1899 | 7.0 years | All stroke | Age, sex, WC, TC, and history of hypertension and DM | 7 |
| Zhang [22] | 2009 | 2002.1 -2008.1 | 2173 | 64.5 | 982/1191 | 5.0 years | IS and HS | Sex, age smoking, drinking and physical activity | 7 |
| The REVEAL-HBV cohort study [23] | 2010 | 1991-2007 | 22472 | 30.0-65.0 | 11391/11081 | 17.0 years | All stroke | HBsAg, age, sex, educational, smoking, alcohol, BMI, TG, TC, DM, hypertension, and CKD | 7 |
| Chen [24] | 2010 | 1990-2006 | 2122 | 55.4 | 854/1268 | 8.7 years | IS | Age | 6 |
| SPRIS [25] | 2011 | 2006.6-2009.6 | 19369 | 52.8 | 10235/9134 | 3.0 years | All stroke | Age, serum TC, current smoking, regular alcohol drinking, atrial fibrillation, family history of CVD | 7 |
| Sun [26] | 2012 | 2009.5-2010.5 | 421 | 60.2 | 341/80 | 1.0 year | All stroke | Age, gender, smoking, DM, hypertension, hyperlipemia, LDL, HDL, TC, TG, creatinine, blood sugar, left ventricular ejection fraction, the time from symptoms onset to balloon dilatation, and the number of stents | 6 |
| Tse [27,28] | 2012 | 1986-2000 | 26607 | >35.0 | 12560/14047 | 10.0 years | IS, HS, and all stroke | Age, study center, history of heart disease or DM, BMI, alcohol drinking habits, alcohol consumption, SBP, years of education and treatment status | 7 |
| EHS [29,30] | 2012 | 1998-2009 | 2137 | 72.0 | 749/1388 | 7.9 years | IS, HS, and all stroke | Age, sex, education, smoking, alcohol drinking and exercise, BMI, TC and mean arterial pressure, baseline CVD history | 8 |
| Zheng [31] | 2012 | 2004-2010 | 3711 | 57.1 | 1588/2123 | 4.9 years | IS and HS | Age, sex, ethnicity, SBP, DBP, BMI, antihypertensive drug use, current smoking, current drinking, DM, TC, HDL, LDL, uric acid, duration of hypertension, and lipid-lowering drug use | 6 |
| Taiwan NHI [32-35] | 2013 | 2000-2003 | 125550 | 42.6 | 38052/87498 | 2.0 years | IS and HS | Age, sex, hypertension, DM, hyperlipidemia, CHD, and the use of anticoagulant medication | 7 |
| Guo [36] | 2013 | 2007.11.1-2012.7.31 | 617 | 78.0 | NA | 2.0 years | IS | Age, gender, hypertension, DM, heart failure, vascular disease, and prior stroke/TIA, warfarin, statin, and diuretic | 6 |
| TwSHHH [37] | 2014 | 2002-2007 | 6331 | 42.2 | 1212/5119 | 4.5 years | IS and HS | Age, sex, smoking status, drinking status, family history of CVD and other MetS components | 7 |
| He [38] | 2014 | 1976-2011 | 1494 | 60.5 | 961/533 | 18.0 years | IS, HS, and all stroke | Age, sex, marital status, occupation, educational level, alcohol drinking, DBP, TG and TC, and BMI | 7 |
| SCH study [39] | 2014 | 1993-2011 | 63257 | 56.5 | 27954/35303 | 14.7 years | IS, HS, and all stroke | Age at recruitment, year of recruitment, sex, dialect, education, BMI, alcohol drinking, smoking, moderate activity, energy intake, dietary intakes of vegetables, fruits, fiber, and polyunsaturated fatty acids | 8 |
| The Chinese AF registry [40] | 2014 | 2008.11-2012.10 | 2016 | 68.5 | 912/1104 | 1.0 year | All stroke | Age, BMI, CVD, hypertension, DM, COPD | 6 |
| SWHS and SMHS [41-48] | 2015 | 1996.10—2013.12 | 113138 | 53.5 | 44590/68548 | 6.1 years for men and 7.1 years for women | IS, HS, and all stroke | Education, income, smoking, alcohol consumption, tea consumption, sex, history of night-shift work, PA, BMI, and waist-to-hip ratio | 8 |
| Han [49] | 2015 | 2010.4-2013.4 | 5488 | > 20.0 | 2712/2776 | 2.7 years | IS | Age, sex, education, smoking, alcohol consumption, BMI, PA, DM, depression, family history of stroke, years of hypertension, antihypertensive medication, and use of folic acid | 7 |
| The SCMS Program [50] | 2015 | 1999.6-2002.11 | 4124 | 74.0 | 2284/1840 | NA | All stroke | DM, hypertension, and age | 7 |
| Zhang [51] | 2015 | 2005.3-2008.4 | 1793 | NA | NA | 5.0 years | All stroke | Baseline characteristics | 6 |
| Zhu [52-59] | 2017 | 2002.6-2012.7 | 2544 | 46.5 | 1042/1502 | 9.2 years | IS and all stroke | Age, sex, smoking, and drinking status, hypertension, family history of CVD, BMI, WC, and levels of blood glucose, TG, LDL, HDL | 8 |
| CSPPT [60-63] | 2017 | 2008.5.19-2013.8.24 | 17720 | 59.9 | 7307/10413 | 4.5 years | IS, HS, and all stroke | Age, sex, study centers, study treatment groups, MTHFR C677T polymorphism, BMI, smoking, SBP, DBP, eGFR levels, fasting glucose, TC, HDL, TG, folate, and homocysteine levels | 8 |
| Han [64] | 2017 | 2001-2011 | 3336 | >60.0 | 1375/1477 | 10.0 years | All stroke | Age, sex, WC, smoking, alcohol consumption, rural-urban living, educational level, marital status, living status, frequency of visiting children/other, help available when needed, hypercholesterolemia, CVD, DM, activities of daily living, depression and dementia | 6 |
| The China Kadoorie Biobank [65-69] | 2018 | 2004.6-2014.9 | 489301 | 51.1 | 200124/289177 | 9.0 years | IS, HS, and all stroke | Age, sex, study area, education, smoking, alcohol consumption, PA, and self-rated health status | 8 |
| LGPT [70] | 2018 | 1986-2014 | 29584 | 51.3 | 13125/16314 | 30.0 years | All stroke | Age at baseline, sex, BMI, commune, education level, tobacco smoking, alcohol drinking, family history of cancer, pulse rate and any drug intake | 8 |
| The Kailuan study [71-84] | 2018 | 2006-2015 | 96110 | 55.6 | 76267/19843 | 8.5 years | IS, HS, and all stroke | Age, sex, smoking, alcohol intake, education, physical activity, average monthly income of each family member, and sodium intake | 8 |
| Tan [85] | 2018 | 1990-2005 | 213221 | 54.7 | 213221/0 | 15.0 years | All stroke | Age, individual-level covariates, and urban/rural, region | 6 |
| China Multi-Provincial Cohort [86] | 2018 | 1992-2012 | 20,954 | 35-64 | 10789/10165 | 20.0 years | HS | Age, sex, BMI, smoking status, alcohol drinking status, family history of CVD, FBG, low HDL-C status, and lipid-lowering  medication | 7 |
| The OSA-ACS project [87] | 2019 | 2015.5-2017.6 | 752 | 57.2 | 621/131 | 1.0 year | All stroke | Crude | 6 |
| Zheng [88] | 2019 | 2004.10-2014.10 | 5097 | 56.3 | 2231/2866 | 8.4 years | IS, HS, and all stroke | Age, sex, ethnicity, BMI, current smoking, heavy drinking, DM, SBP, DBP, and anti-hypertension drug treatment | 7 |
| Gu [89] | 2019 | 1992-2015 | 267500* | 50.4 | 159478/108022 | 8.6 years | IS and HS | Sex, age, smoking status, hypertension, geographic region, alcohol consumption, education level, and BMI | 7 |

#### BMI: body mass index; CHD: coronary heart disease; CKD: chronic kidney disease; COPD: chronic obstructive pulmonary disease; CVD: cardiovascular disease; DBP: [diastolic](file:///C:/Program%2520Files%2520(x86)/Youdao/Dict/8.5.3.0/resultui/html/index.html#/javascript:;) [blood](file:///C:/Program%2520Files%2520(x86)/Youdao/Dict/8.5.3.0/resultui/html/index.html#/javascript:;) [pressure](file:///C:/Program%2520Files%2520(x86)/Youdao/Dict/8.5.3.0/resultui/html/index.html#/javascript:;); DM: diabetes mellitus; eGFR: estimated [glomerular filtration rate](file:///C:/Program%2520Files%2520(x86)/Youdao/Dict/8.5.3.0/resultui/html/index.html#/javascript:;); HDL: high density lipoprotein; HS: hemorrhagic stroke; IS: ischemic stroke; LDL: [low](file:///C:/Program%2520Files%2520(x86)/Youdao/Dict/8.5.3.0/resultui/html/index.html#/javascript:;) [density](file:///C:/Program%2520Files%2520(x86)/Youdao/Dict/8.5.3.0/resultui/html/index.html#/javascript:;) [lipoprotein](file:///C:/Program%2520Files%2520(x86)/Youdao/Dict/8.5.3.0/resultui/html/index.html#/javascript:;); PA: physical activity; SBP: [systolic](file:///C:/Program%2520Files%2520(x86)/Youdao/Dict/8.5.3.0/resultui/html/index.html#/javascript:;) blood [pressure](file:///C:/Program%2520Files%2520(x86)/Youdao/Dict/8.5.3.0/resultui/html/index.html#/javascript:;); WC: [wrist circumference](file:///C:/Program%2520Files%2520(x86)/Youdao/Dict/8.5.3.0/resultui/html/index.html#/javascript:;); TC: total cholesterol; TG: [triglyceride](file:///C:/Program%2520Files%2520(x86)/Youdao/Dict/8.5.3.0/resultui/html/index.html#/javascript:;);

#### *This study specifically contained the Kailuan study cohort.

**Reference:**

1. Yuan JM, Ross RK, Wang XL, Gao YT, Henderson BE, Yu MC. Morbidity and mortality in relation to cigarette smoking in Shanghai, China. A prospective male cohort study. *Jama.* 1996;275(21):1646-1650.
2. Hsu HC, Pwu RF. Too late to quit? Effect of smoking and smoking cessation on morbidity and mortality among the elderly in a longitudinal study. *The Kaohsiung journal of medical sciences.* 2004;20(10):484-491.
3. Zhang XF, Attia J, D'Este C, Yu XH. Prevalence and magnitude of classical risk factors for stroke in a cohort of 5092 Chinese steelworkers over 13.5 years of follow-up. *Stroke.* 2004;35(5):1052-1056.
4. Chen HJ, Bai CH, Yeh WT, Chiu HC, Pan WH. Influence of metabolic syndrome and general obesity on the risk of ischemic stroke. *Stroke.* 2006;37(4):1060-1064.
5. Chuang SY, Bai CH, Chen WH, Lien LM, Pan WH. Fibrinogen independently predicts the development of ischemic stroke in a Taiwanese population: CVDFACTS study. *Stroke.* 2009;40(5):1578-1584.
6. Fang XH, Zhang XH, Yang QD, et al. Subtype hypertension and risk of stroke in middle-aged and older Chinese: a 10-year follow-up study. *Stroke.* 2006;37(1):38-43.
7. Bazzano LA, Gu D, Reynolds K, et al. Alcohol consumption and risk for stroke among Chinese men. *Annals of neurology.* 2007;62(6):569-578.
8. Bazzano LA, Gu D, Whelton MR, et al. Body mass index and risk of stroke among Chinese men and women. *Annals of neurology.* 2010;67(1):11-20.
9. Gu D, Kelly TN, Wu X, et al. Blood pressure and risk of cardiovascular disease in Chinese men and women. *American journal of hypertension.* 2008;21(3):265-272.
10. Kelly TN, Gu D, Chen J, et al. Cigarette smoking and risk of stroke in the chinese adult population. *Stroke.* 2008;39(6):1688-1693.
11. Chen HK, Tseng CD, Wu SC, Lee TK, Chen TH. A prospective cohort study on the effect of sexual activity, libido and widowhood on mortality among the elderly people: 14-year follow-up of 2,453 elderly Taiwanese. *International journal of epidemiology.* 2007;36(5):1136-1142.
12. Yang X, Ko GT, So WY, et al. Additive interaction of hyperglycemia and albuminuria on risk of ischemic stroke in type 2 diabetes: Hong Kong Diabetes Registry. *Diabetes care.* 2008;31(12):2294-2300.
13. Yang X, So WY, Ma RC, et al. Thresholds of risk factors for ischemic stroke in type 2 diabetic patients with and without albuminuria: a non-linear approach. *Clinical neurology and neurosurgery.* 2008;110(7):701-709.
14. Zhou M, Offer A, Yang G, et al. Body mass index, blood pressure, and mortality from stroke: a nationally representative prospective study of 212,000 Chinese men. *Stroke.* 2008;39(3):753-759.
15. Chen JH, Chuang SY, Chen HJ, Yeh WT, Pan WH. Serum uric acid level as an independent risk factor for all-cause, cardiovascular, and ischemic stroke mortality: a Chinese cohort study. *Arthritis and rheumatism.* 2009;61(2):225-232.
16. Chien KL, Sung FC, Hsu HC, Su TC, Lin RS, Lee YT. Apolipoprotein A-I and B and stroke events in a community-based cohort in Taiwan: report of the Chin-Shan Community Cardiovascular Study. *Stroke.* 2002;33(1):39-44.
17. Chen PC, Chien KL, Hsu HC, Su TC, Sung FC, Lee YT. Metabolic syndrome and C-reactive protein in stroke prediction: a prospective study in Taiwan. *Metabolism: clinical and experimental.* 2009;58(6):772-778.
18. Chien KL, Hsu HC, Sung FC, Su TC, Chen MF, Lee YT. Metabolic syndrome as a risk factor for coronary heart disease and stroke: an 11-year prospective cohort in Taiwan community. *Atherosclerosis.* 2007;194(1):214-221.
19. Chien KL, Hsu HC, Su TC, Sung FC, Chen MF, Lee YT. Lipoprotein(a) and cardiovascular disease in ethnic Chinese: the Chin-Shan Community Cardiovascular Cohort Study. *Clinical chemistry.* 2008;54(2):285-291.
20. Chien KL, Su TC, Hsu HC, et al. Atrial fibrillation prevalence, incidence and risk of stroke and all-cause death among Chinese. *International journal of cardiology.* 2010;139(2):173-180.
21. Sun Y, Chien KL, Hsu HC, Su TC, Chen MF, Lee YT. Use of serum homocysteine to predict stroke, coronary heart disease and death in ethnic Chinese. 12-year prospective cohort study. *Circulation journal : official journal of the Japanese Circulation Society.* 2009;73(8):1423-1430.
22. Zhang WW, Liu CY, Wang YJ, Xu ZQ, Chen Y, Zhou HD. Metabolic syndrome increases the risk of stroke: a 5-year follow-up study in a Chinese population. *Journal of neurology.* 2009;256(9):1493-1499.
23. Wang CH, Chen CJ, Lee MH, Yang HI, Hsiao CK. Chronic hepatitis B infection and risk of atherosclerosis-related mortality: A 17-year follow-up study based on 22,472 residents in Taiwan. *Atherosclerosis.* 2010;211(2):624-629.
24. Chen XY, Thomas GN, Chen YK, Chan JC, Wong KS. Atherosclerotic vascular disease rather than metabolic syndrome predicts ischemic stroke in diabetic patients. *Cerebrovascular diseases.* 2010;30(4):374-379.
25. Jia Z, Wu S, Zhou Y, et al. Metabolic syndrome and its components as predictors of stroke in middle-aged and elderly Chinese people. *Neurological research.* 2011;33(5):453-459.
26. Sun Y, Jiang D, Zhang B, et al. Impact of obesity on the outcome of Chinese patients with ST-segment myocardial infarction undergoing urgent percutaneous coronary intervention. *Acta cardiologica.* 2012;67(5):541-548.
27. Tse LA, Fang XH, Wang WZ, Qiu H, Yu IT. Incidence of ischaemic and haemorrhagic stroke and the association with smoking and smoking cessation: a 10-year multicentre prospective study in China. *Public health.* 2012;126(11):960-966.
28. Wang C, Liu Y, Yang Q, et al. Body mass index and risk of total and type-specific stroke in Chinese adults: results from a longitudinal study in China. *International journal of stroke : official journal of the International Stroke Society.* 2013;8(4):245-250.
29. Xu L, Chan WM, Hui YF, Lam TH. Association between HbA1c and cardiovascular disease mortality in older Hong Kong Chinese with diabetes. *Diabetic medicine : a journal of the British Diabetic Association.* 2012;29(3):393-398.
30. Xu L, Schooling CM, Chan WM, Lee SY, Leung GM, Lam TH. Smoking and hemorrhagic stroke mortality in a prospective cohort study of older Chinese. *Stroke.* 2013;44(8):2144-2149.
31. Zheng L, Sun Z, Zhang X, Li J, Hu D, Sun Y. The association between glomerular filtration rate and stroke in hypertensive patients in rural areas of China. *Journal of hypertension.* 2012;30(5):901-907.
32. Kuo CY, Yen MF, Chen LS, et al. Increased risk of hemorrhagic stroke in patients with migraine: a population-based cohort study. *PloS one.* 2013;8(1):e55253.
33. Wang TJ, Keller JJ, Sheu JJ, Lin HC. A 3-year follow-up study on the risk of stroke among patients with conjunctival haemorrhage. *Acta ophthalmologica.* 2013;91(3):226-230.
34. Wu CH, Liou TH, Chen HH, Sun TY, Chen KH, Chang KH. Stroke risk in poliomyelitis survivors: a nationwide population-based study. *Archives of physical medicine and rehabilitation.* 2012;93(12):2184-2188.
35. Wu CW, Chao PZ, Hao WR, Liou TH, Lin HW. Risk of stroke among patients with rhinosinusitis: a population-based study in Taiwan. *American journal of rhinology & allergy.* 2012;26(4):278-282.
36. Guo Y, Wang H, Zhao X, et al. Sequential changes in renal function and the risk of stroke and death in patients with atrial fibrillation. *International journal of cardiology.* 2013;168(5):4678-4684.
37. Chen YC, Sun CA, Yang T, et al. Impact of metabolic syndrome components on incident stroke subtypes: a Chinese cohort study. *Journal of human hypertension.* 2014;28(11):689-693.
38. He Y, Jiang B, Li LS, et al. Changes in smoking behavior and subsequent mortality risk during a 35-year follow-up of a cohort in Xi'an, China. *American journal of epidemiology.* 2014;179(9):1060-1070.
39. Pan A, De Silva DA, Yuan JM, Koh WP. Sleep duration and risk of stroke mortality among Chinese adults: Singapore Chinese health study. *Stroke.* 2014;45(6):1620-1625.
40. Yang YM, Shao XH, Zhu J, et al. Risk factors and incidence of stroke and MACE in Chinese atrial fibrillation patients presenting to emergency departments: a national wide database analysis. *International journal of cardiology.* 2014;173(2):242-247.
41. Cai H, Shu XO, Xiang YB, et al. Sleep duration and mortality: a prospective study of 113 138 middle-aged and elderly Chinese men and women. *Sleep.* 2015;38(4):529-536.
42. Yu D, Zhang X, Shu XO, et al. Dietary glycemic index, glycemic load, and refined carbohydrates are associated with risk of stroke: a prospective cohort study in urban Chinese women. *The American journal of clinical nutrition.* 2016;104(5):1345-1351.
43. Cai H, Shu XO, Gao YT, Li H, Yang G, Zheng W. A prospective study of dietary patterns and mortality in Chinese women. *Epidemiology.* 2007;18(3):393-401.
44. Dorjgochoo T, Shu XO, Zhang X, et al. Relation of blood pressure components and categories and all-cause, stroke and coronary heart disease mortality in urban Chinese women: a population-based prospective study. *Journal of hypertension.* 2009;27(3):468-475.
45. Takata Y, Shu XO, Gao YT, et al. Red meat and poultry intakes and risk of total and cause-specific mortality: results from cohort studies of Chinese adults in Shanghai. *PloS one.* 2013;8(2):e56963.
46. Takata Y, Zhang X, Li H, et al. Fish intake and risks of total and cause-specific mortality in 2 population-based cohort studies of 134,296 men and women. *American journal of epidemiology.* 2013;178(1):46-57.
47. Yu D, Shu XO, Li H, et al. Dietary isoflavones, urinary isoflavonoids, and risk of ischemic stroke in women. *The American journal of clinical nutrition.* 2015;102(3):680-686.
48. Zhang X, Shu XO, Gao YT, Yang G, Li H, Zheng W. General and abdominal adiposity and risk of stroke in Chinese women. *Stroke.* 2009;40(4):1098-1104.
49. Han L, Wu Q, Wang C, et al. Homocysteine, Ischemic Stroke, and Coronary Heart Disease in Hypertensive Patients: A Population-Based, Prospective Cohort Study. *Stroke.* 2015;46(7):1777-1786.
50. Lai YJ, Chen HC, Chou P. Gender Difference in the Interaction Effects of Diabetes and Hypertension on Stroke among the Elderly in the Shih-Pai Study, Taiwan. *PloS one.* 2015;10(8):e0136634.
51. Zhang YJ, Iqbal J, van Klaveren D, et al. Smoking is associated with adverse clinical outcomes in patients undergoing revascularization with PCI or CABG: the SYNTAX trial at 5-year follow-up. *Journal of the American College of Cardiology.* 2015;65(11):1107-1115.
52. Zhu ZB, Huangfu XF, Zhong CK, et al. Combined Effects of Family History of Cardiovascular Disease and Serum C-reactive Protein Level on the Risk of Stroke: A 9.2-year Prospective Study among Mongolians in China. *Biomedical and environmental sciences : BES.* 2017;30(9):632-640.
53. Huangfu X, Zhu Z, Zhong C, et al. Smoking, Hypertension, and Their Combined Effect on Ischemic Stroke Incidence: A Prospective Study among Inner Mongolians in China. *Journal of stroke and cerebrovascular diseases : the official journal of National Stroke Association.* 2017;26(12):2749-2754.
54. Xu J, Xu T, Bu X, et al. The predictive value of waist-to-height ratio for ischemic stroke in a population-based prospective cohort study among Mongolian men in China. *PloS one.* 2014;9(10):e110245.
55. Olofindayo J, Peng H, Liu Y, et al. The interactive effect of diabetes and central obesity on stroke: a prospective cohort study of inner Mongolians. *BMC neurology.* 2015;15:65.
56. Tang L, Xu T, Li H, et al. Hypertension, alcohol drinking and stroke incidence: a population-based prospective cohort study among inner Mongolians in China. *Journal of hypertension.* 2014;32(5):1091-1096; discussion 1096.
57. Wang A, Xu T, Xu T, et al. Hypertension and elevated C-reactive protein: future risk of ischemic stroke in a prospective cohort study among inner Mongolians in China. *International journal of cardiology.* 2014;174(2):455-456.
58. Xu T, Bu X, Li H, et al. Smoking, heart rate, and ischemic stroke: a population-based prospective cohort study among Inner Mongolians in China. *Stroke.* 2013;44(9):2457-2461.
59. Zhong C, Zhong X, Xu T, et al. Combined effects of hypertension and heart rate on the risk of stroke and coronary heart disease: a population-based prospective cohort study among Inner Mongolians in China. *Hypertension research : official journal of the Japanese Society of Hypertension.* 2015;38(12):883-888.
60. Fan F, Yuan Z, Qin X, et al. Optimal Systolic Blood Pressure Levels for Primary Prevention of Stroke in General Hypertensive Adults: Findings From the CSPPT (China Stroke Primary Prevention Trial). *Hypertension.* 2017;69(4):697-704.
61. Shi X, Yang J, Wang L, et al. Prospective study of serum uric acid levels and stroke in a Chinese hypertensive cohort. *Clinical and experimental hypertension.* 2017;39(6):527-531.
62. Xu RB, Kong X, Xu BP, et al. Longitudinal association between fasting blood glucose concentrations and first stroke in hypertensive adults in China: effect of folic acid intervention. *The American journal of clinical nutrition.* 2017;105(3):564-570.
63. Zhang C, Wang X, He M, et al. Proteinuria Is an Independent Risk Factor for First Incident Stroke in Adults Under Treatment for Hypertension in China. *Journal of the American Heart Association.* 2015;4(12).
64. Han TS, Wang HH, Wei L, et al. Impacts of undetected and inadequately treated hypertension on incident stroke in China. *BMJ open.* 2017;7(10):e016581.
65. Chen Z, Iona A, Parish S, et al. Adiposity and risk of ischaemic and haemorrhagic stroke in 0.5 million Chinese men and women: a prospective cohort study. *The Lancet Global health.* 2018;6(6):e630-e640.
66. Du H, Li L, Bennett D, et al. Fresh Fruit Consumption and Major Cardiovascular Disease in China. *The New England journal of medicine.* 2016;374(14):1332-1343.
67. Bragg F, Li L, Yang L, et al. Risks and Population Burden of Cardiovascular Diseases Associated with Diabetes in China: A Prospective Study of 0.5 Million Adults. *PLoS medicine.* 2016;13(7):e1002026.
68. Qin C, Lv J, Guo Y, et al. Associations of egg consumption with cardiovascular disease in a cohort study of 0.5 million Chinese adults. *Heart.* 2018;104(21):1756-1763.
69. Sun J, Ma H, Yu C, et al. Association of Major Depressive Episodes With Stroke Risk in a Prospective Study of 0.5 Million Chinese Adults. *Stroke.* 2016;47(9):2203-2208.
70. Fan JH, Wang JB, Wang SM, Abnet CC, Qiao YL, Taylor PR. Longitudinal change in blood pressure is associated with cardiovascular disease mortality in a Chinese cohort. *Heart.* 2018;104(21):1764-1771.
71. Jin C, Li G, Rexrode KM, et al. Prospective Study of Fasting Blood Glucose and Intracerebral Hemorrhagic Risk. *Stroke.* 2018;49(1):27-33.
72. Wu J, Chen S, Zhou Y, et al. Non-high-density lipoprotein cholesterol on the risks of stroke: a result from the Kailuan study. *PloS one.* 2013;8(9):e74634.
73. Liu Y, Wang J, Zhang L, et al. Relationship between C-reactive protein and stroke: a large prospective community based study. *PloS one.* 2014;9(9):e107017.
74. Wang A, Chen S, Wang C, et al. Resting heart rate and risk of cardiovascular diseases and all-cause death: the Kailuan study. *PloS one.* 2014;9(10):e110985.
75. Li Z, Wang A, Cai J, et al. Impact of proteinuria and glomerular filtration rate on risk of ischaemic and intracerebral hemorrhagic stroke: a result from the Kailuan study. *European journal of neurology.* 2015;22(2):355-360.
76. Li W, Jin C, Vaidya A, et al. Blood Pressure Trajectories and the Risk of Intracerebral Hemorrhage and Cerebral Infarction: A Prospective Study. *Hypertension.* 2017;70(3):508-514.
77. Ma C, Pavlova M, Liu Y, et al. Probable REM sleep behavior disorder and risk of stroke: A prospective study. *Neurology.* 2017;88(19):1849-1855.
78. Song Q, Liu X, Zhou W, et al. Long Sleep Duration and Risk of Ischemic Stroke and Hemorrhagic Stroke: the Kailuan Prospective Study. *Scientific reports.* 2016;6:33664.
79. Wang A, Wu J, Zhou Y, et al. Measures of adiposity and risk of stroke in China: a result from the Kailuan study. *PloS one.* 2013;8(4):e61665.
80. Wang A, Liu X, Su Z, et al. Two-Year Changes in Proteinuria and the Risk of Stroke in the Chinese Population: A Prospective Cohort Study. *Journal of the American Heart Association.* 2017;6(7).
81. Wang A, Jiang R, Su Z, et al. Association of Persistent, Incident, and Remittent Proteinuria With Stroke Risk in Patients With Diabetes Mellitus or Prediabetes Mellitus. *Journal of the American Heart Association.* 2017;6(10).
82. Wang A, Liu J, Li C, et al. Cumulative Exposure to High-Sensitivity C-Reactive Protein Predicts the Risk of Cardiovascular Disease. *Journal of the American Heart Association.* 2017;6(10).
83. Wu J, Chen S, Liu L, et al. Non-high-density lipoprotein cholesterol vs low-density lipoprotein cholesterol as a risk factor for ischemic stroke: a result from the Kailuan study. *Neurological research.* 2013;35(5):505-511.
84. Yu J, Dai L, Zhao Q, et al. Association of Cumulative Exposure to Resting Heart Rate with Risk of Stroke in General Population: The Kailuan Cohort Study. *Journal of stroke and cerebrovascular diseases : the official journal of National Stroke Association.* 2017;26(11):2501-2509.
85. Tan J, Zhang X, Wang W, Yin P, Guo X, Zhou M. Smoking, Blood Pressure, and Cardiovascular Disease Mortality in a Large Cohort of Chinese Men with 15 Years Follow-up. *International journal of environmental research and public health.* 2018;15(5).
86. Zhang X, Liu J, Wang M, et al. Twenty-year epidemiologic study on LDL-C levels in relation to the risks of atherosclerotic event, hemorrhagic stroke, and cancer death among young and middle-aged population in China. *Journal of clinical lipidology.* 2018;12(5):1179-1189 e1174.
87. Zeng Y, Yang S, Wang X, Fan J, Nie S, Wei Y. Prognostic impact of residual SYNTAX score in patients with obstructive sleep apnea and acute coronary syndrome: a prospective cohort study. *Respiratory research.* 2019;20(1):43.
88. Zheng J, Sun Z, Zhang X, et al. Non-traditional lipid profiles associated with ischemic stroke not hemorrhagic stroke in hypertensive patients: results from an 8.4 years follow-up study. *Lipids in health and disease.* 2019;18(1):9.
89. Gu X, Li Y, Chen S, et al. Association of Lipids With Ischemic and Hemorrhagic Stroke: A Prospective Cohort Study Among 267 500 Chinese. *Stroke.* 2019;50(12):3376-3384.
