# Supplementary material for: Identification of Risk Factors for Stroke in China: A Meta-Analysis of Prospective Cohort Studies
Source: Front Neurol. 2022 Mar 18;13:847304. doi: 10.3389/fneur.2022.847304 (PMC8972128; doi:10.3389/fneur.2022.847304)
Supplement: Supplementary file 3 [file Table_3.DOCX]

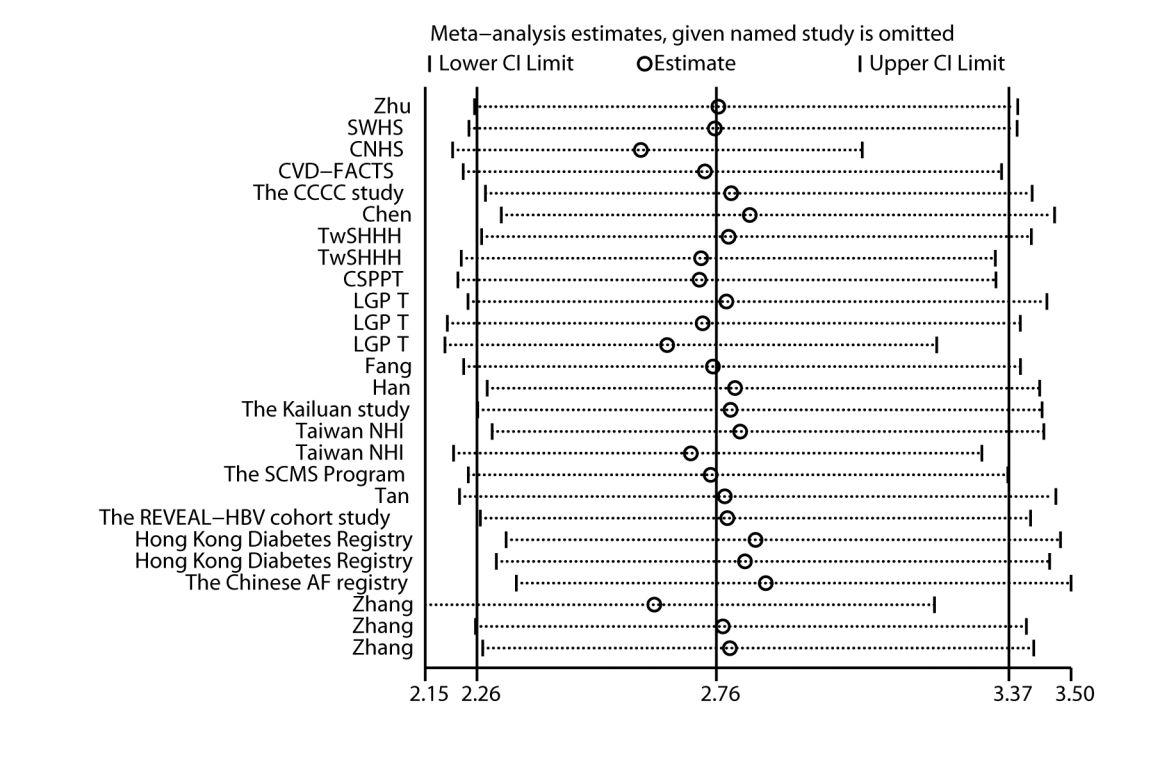


Figure S1. Sensitivity analysis for hypertension


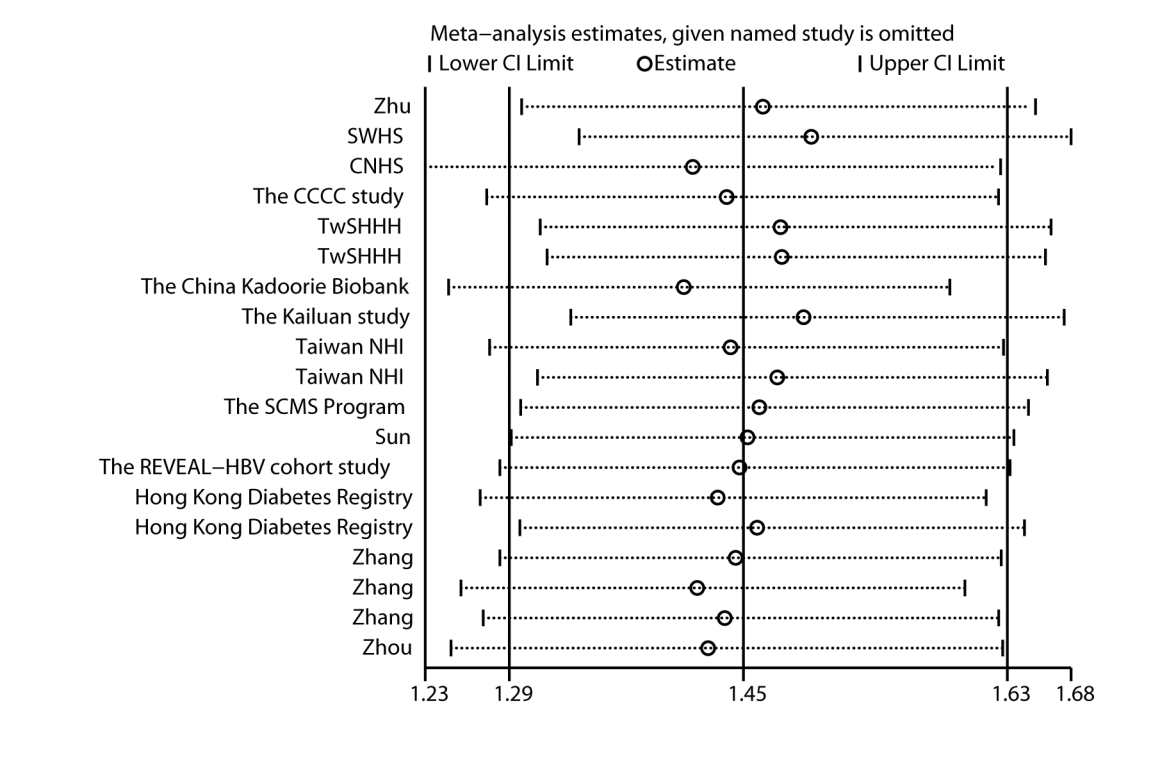


Figure S2. Sensitivity analysis for obesity


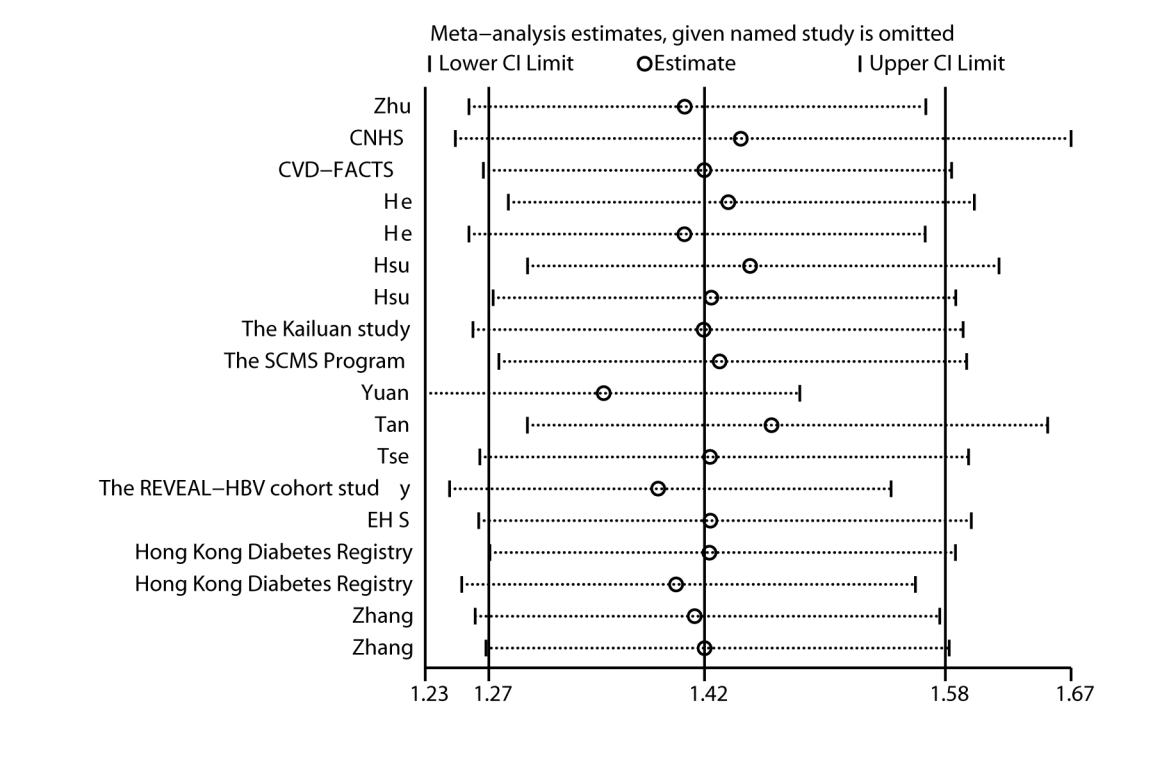


Figure S3. Sensitivity analysis for smoking
