# Supplementary material for: Identification of Risk Factors for Stroke in China: A Meta-Analysis of Prospective Cohort Studies
Source: Front Neurol. 2022 Mar 18;13:847304. doi: 10.3389/fneur.2022.847304 (PMC8972128; doi:10.3389/fneur.2022.847304)
Supplement: Supplementary file 4 [file Table_4.DOCX]

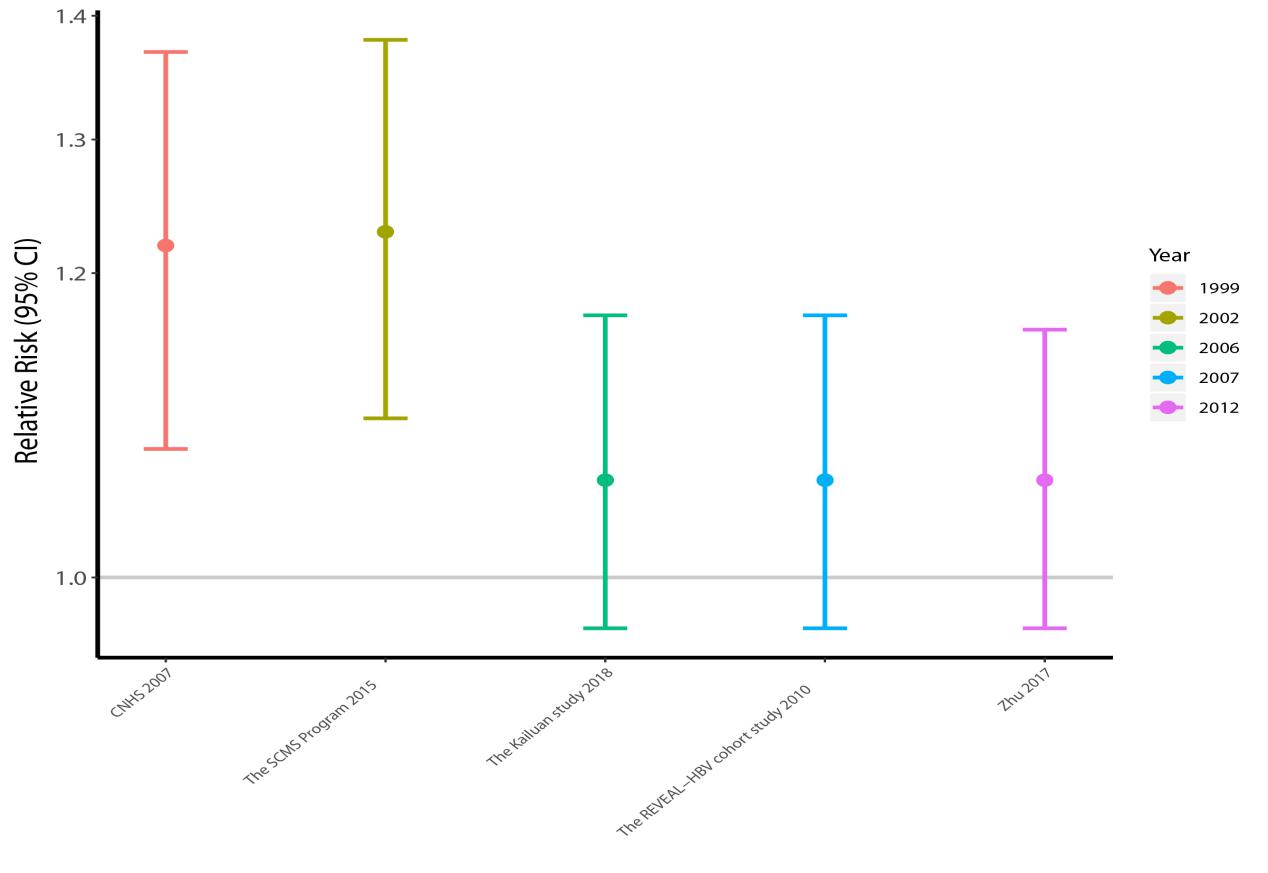


Figure S1. The role of cohort inclusion years for the association between alcohol intake and all stroke risk (*P*=0.096)


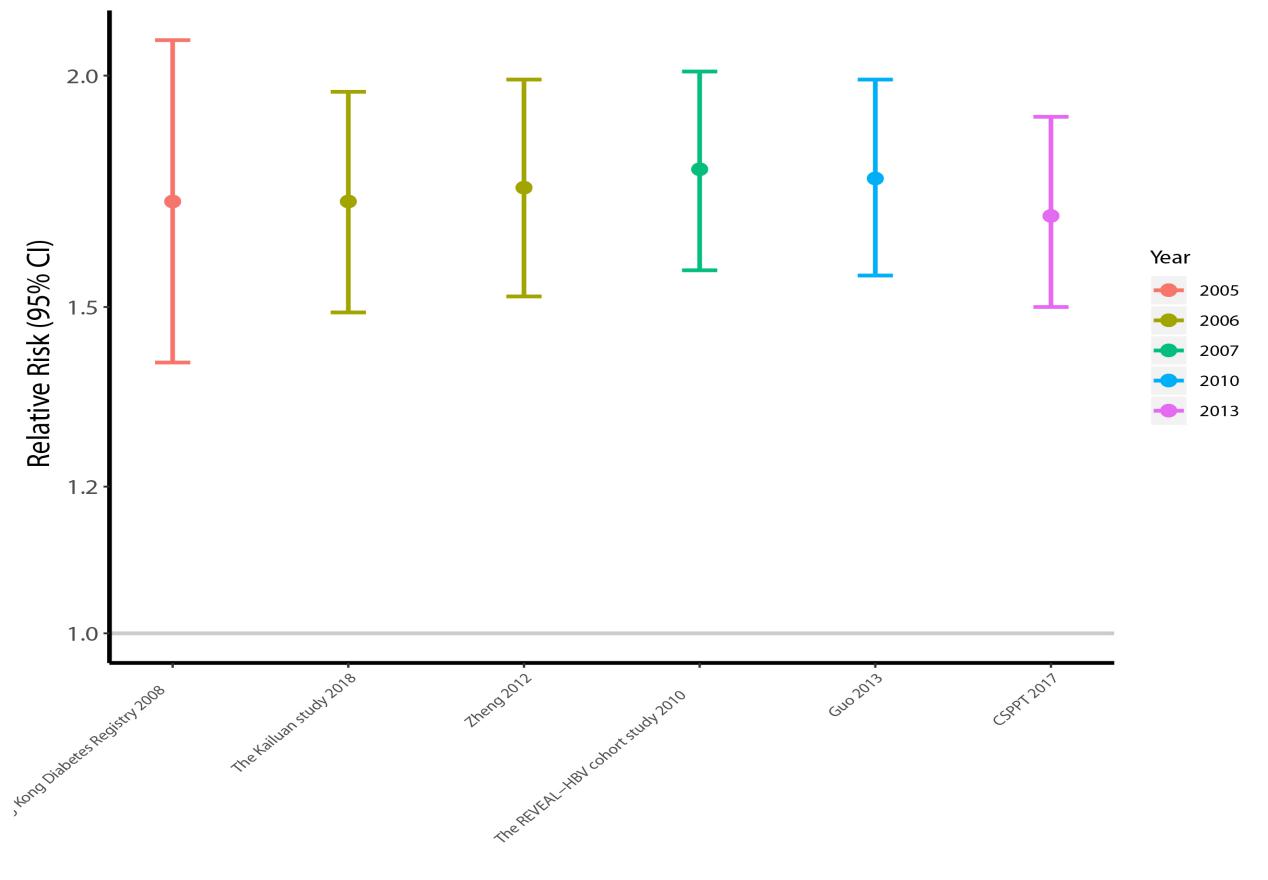


Figure S2. The role of cohort inclusion years for the association between CKD and all stroke risk (*P*=0.760)


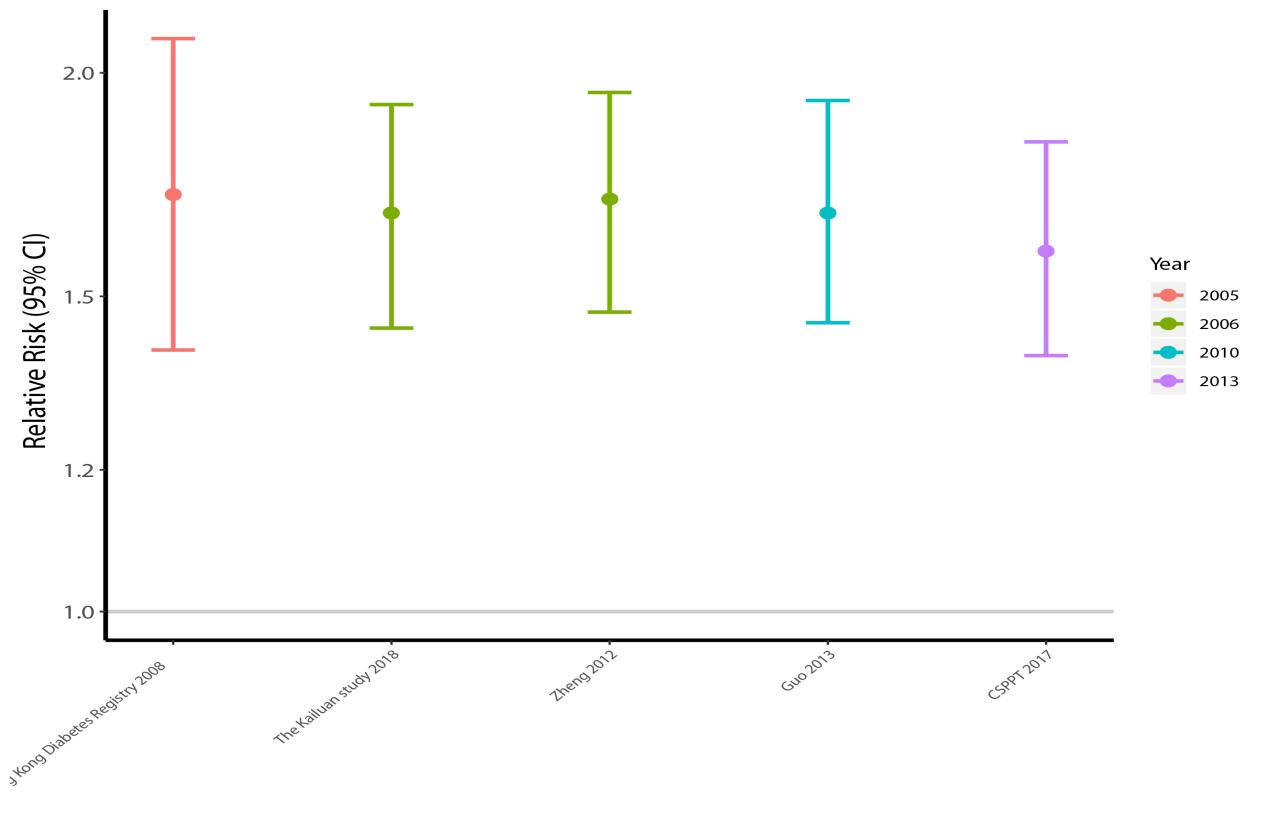


Figure S3. The role of cohort inclusion years for the association between CKD and IS risk (*P*=0.541)


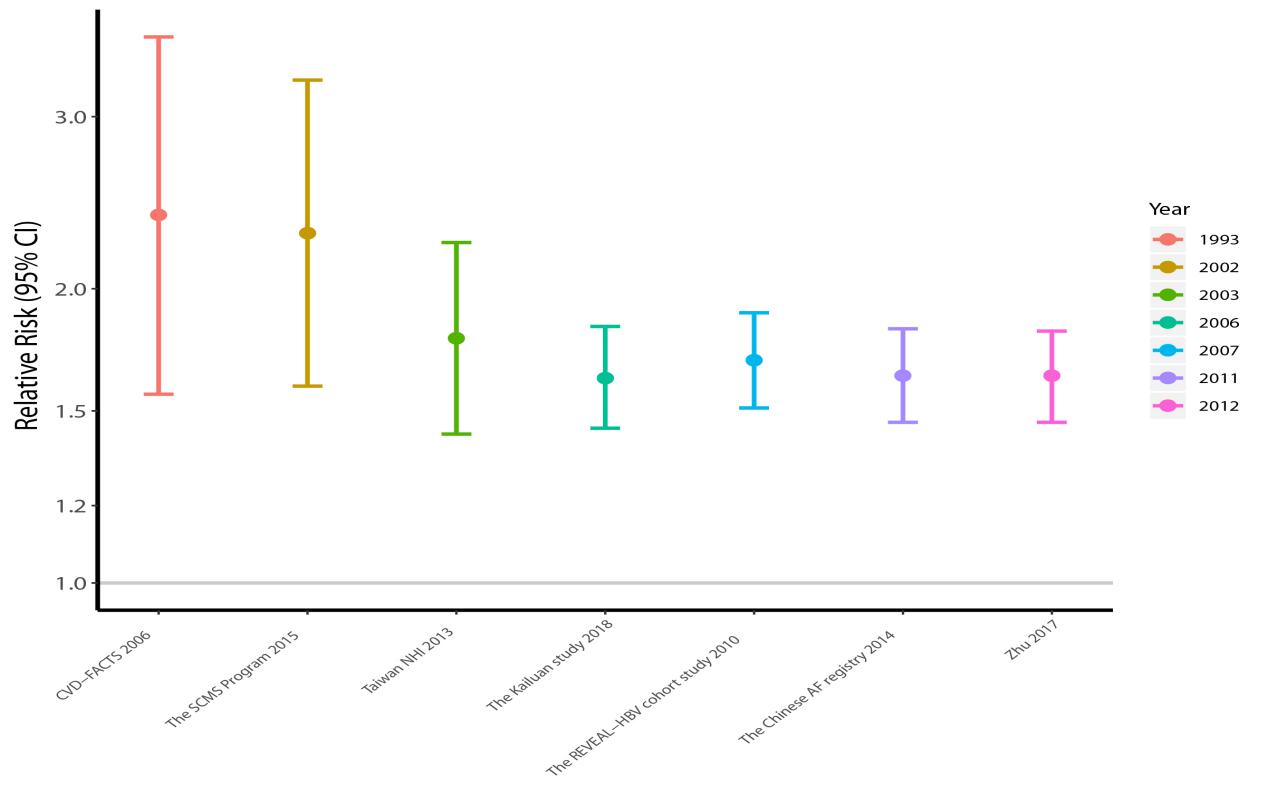


Figure S4. The role of cohort inclusion years for the association between DM and all stroke risk (*P*=0.129)


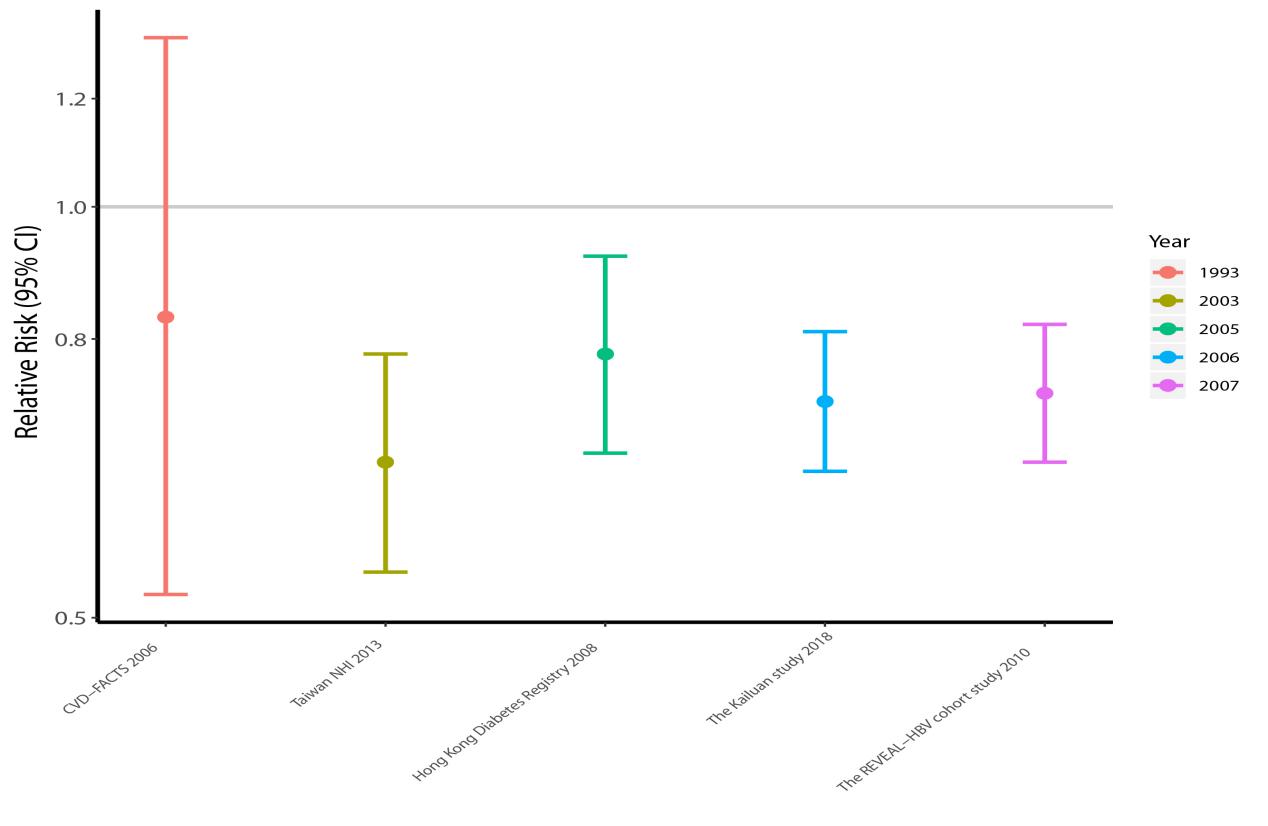


Figure S5. The role of cohort inclusion years for the association between sex difference and all stroke risk (*P*=0.982)


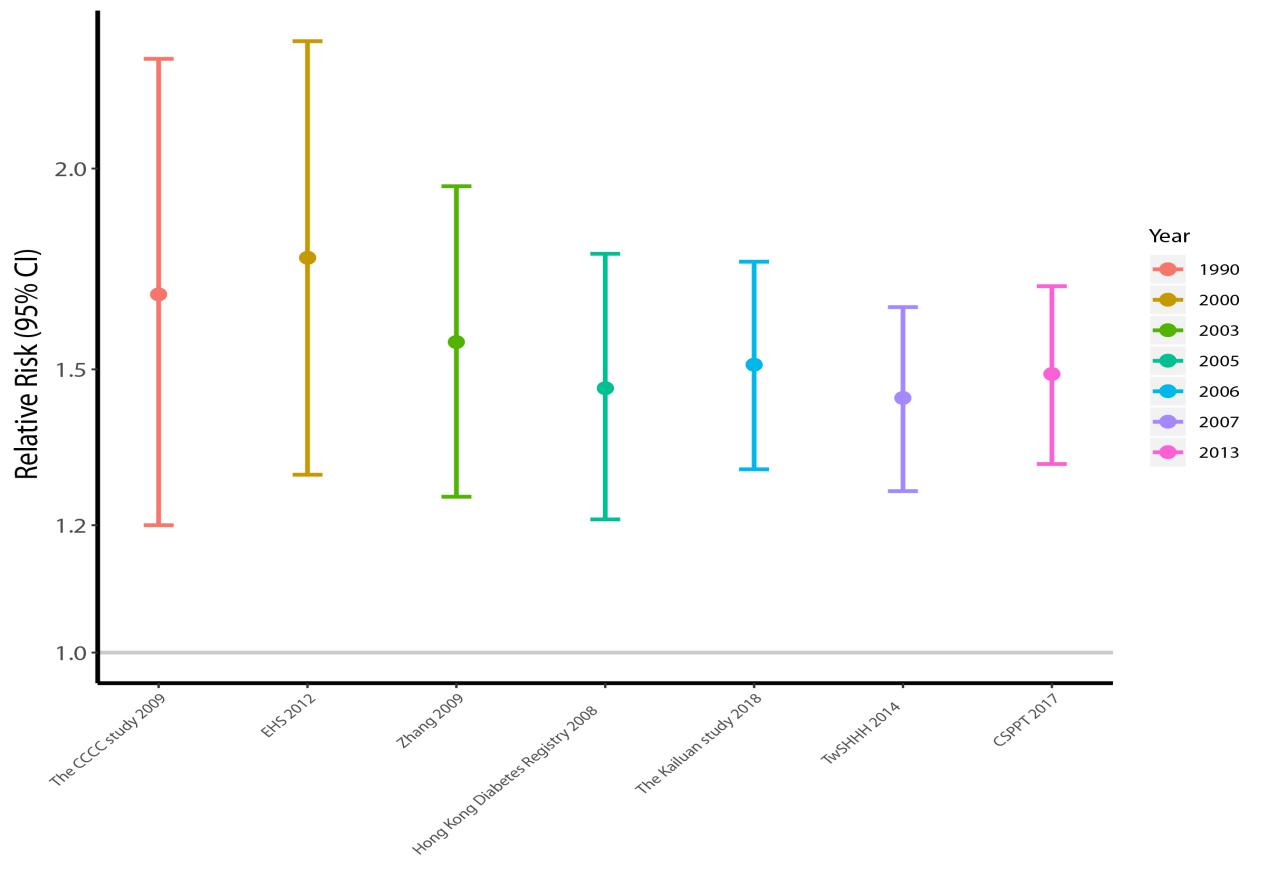


Figure S6. The role of cohort inclusion years for the association between hyperglycemia and all stroke risk (*P*=0.442)


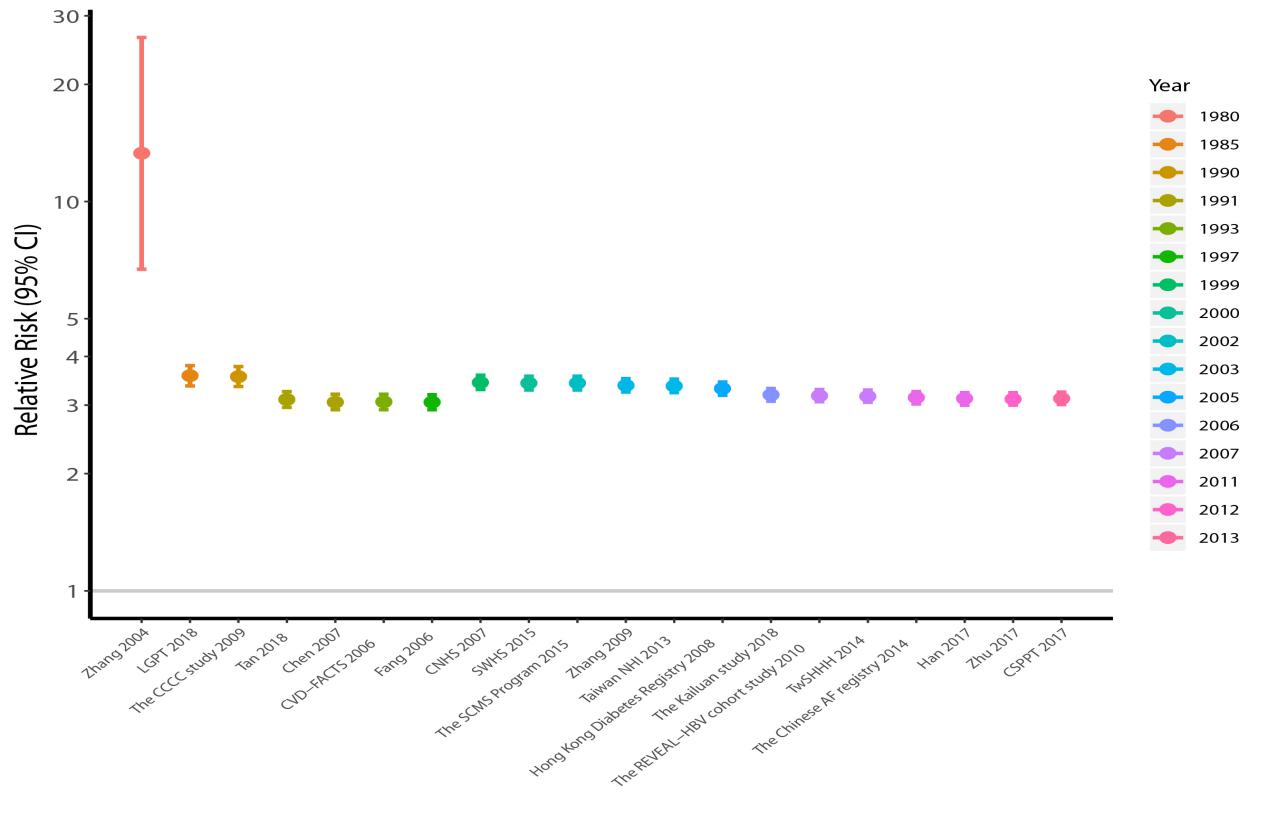


Figure S7. The role of cohort inclusion years for the association between hypertension and all stroke risk (*P*=0.211)


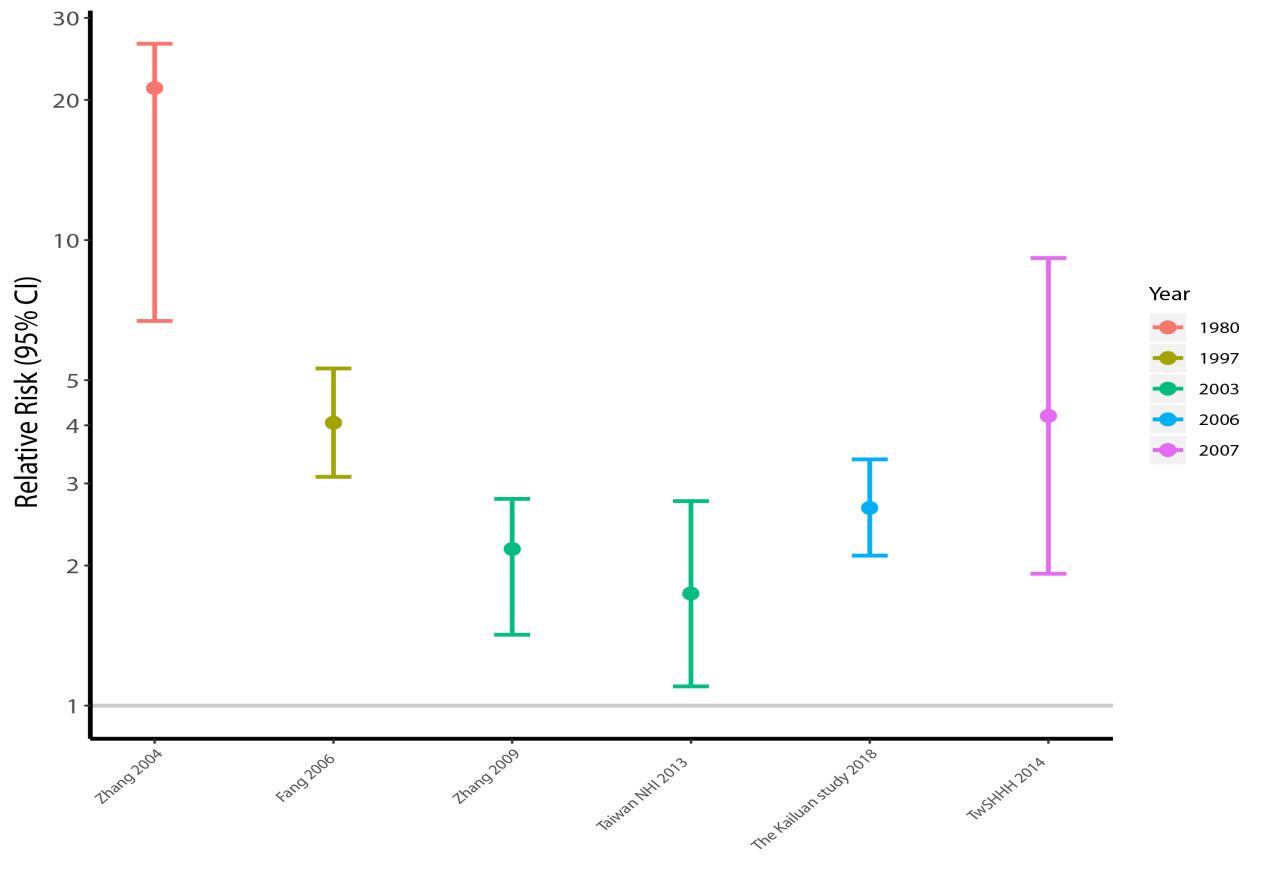


Figure S8. The role of cohort inclusion years for the association between hypertension and HS risk (*P*=0.020)


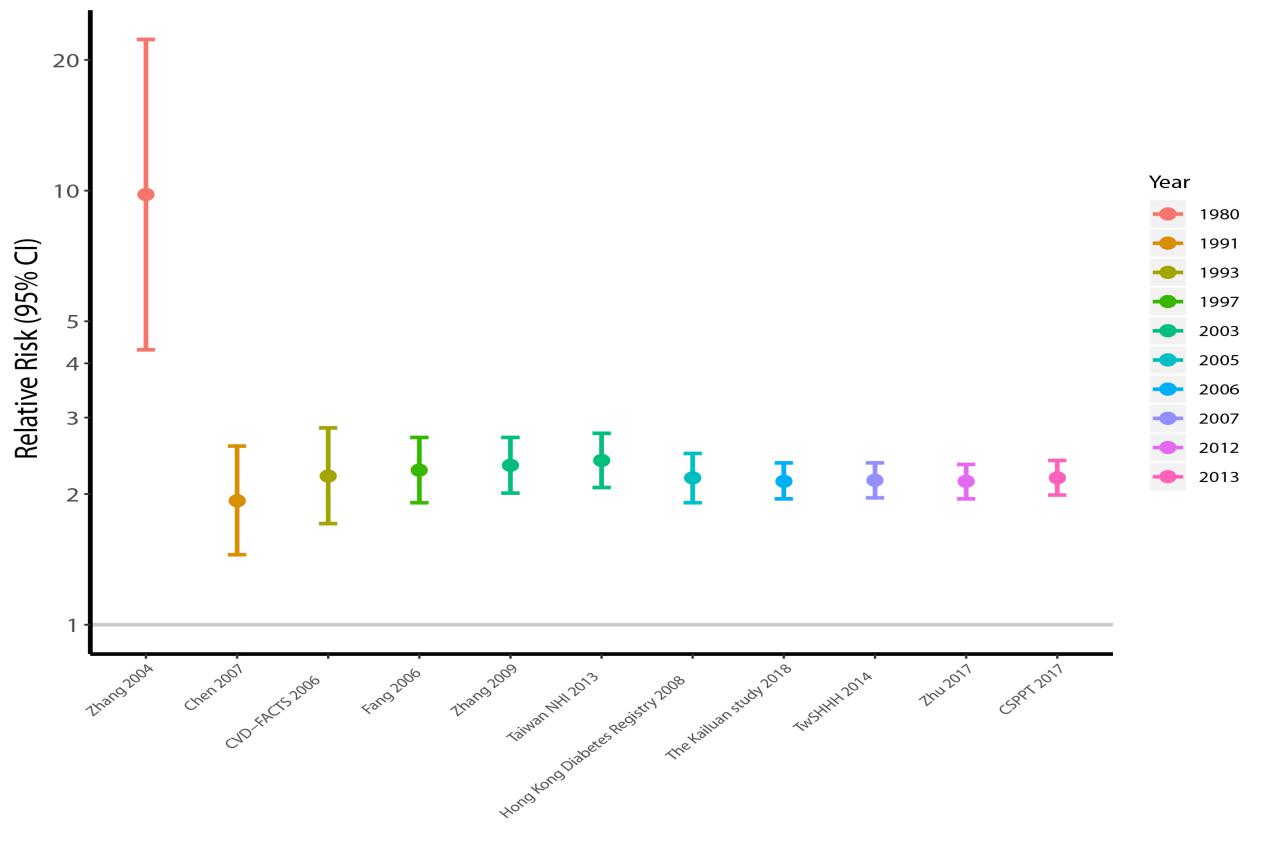


Figure S9. The role of cohort inclusion years for the association between hypertension and IS risk (*P*=0.349)


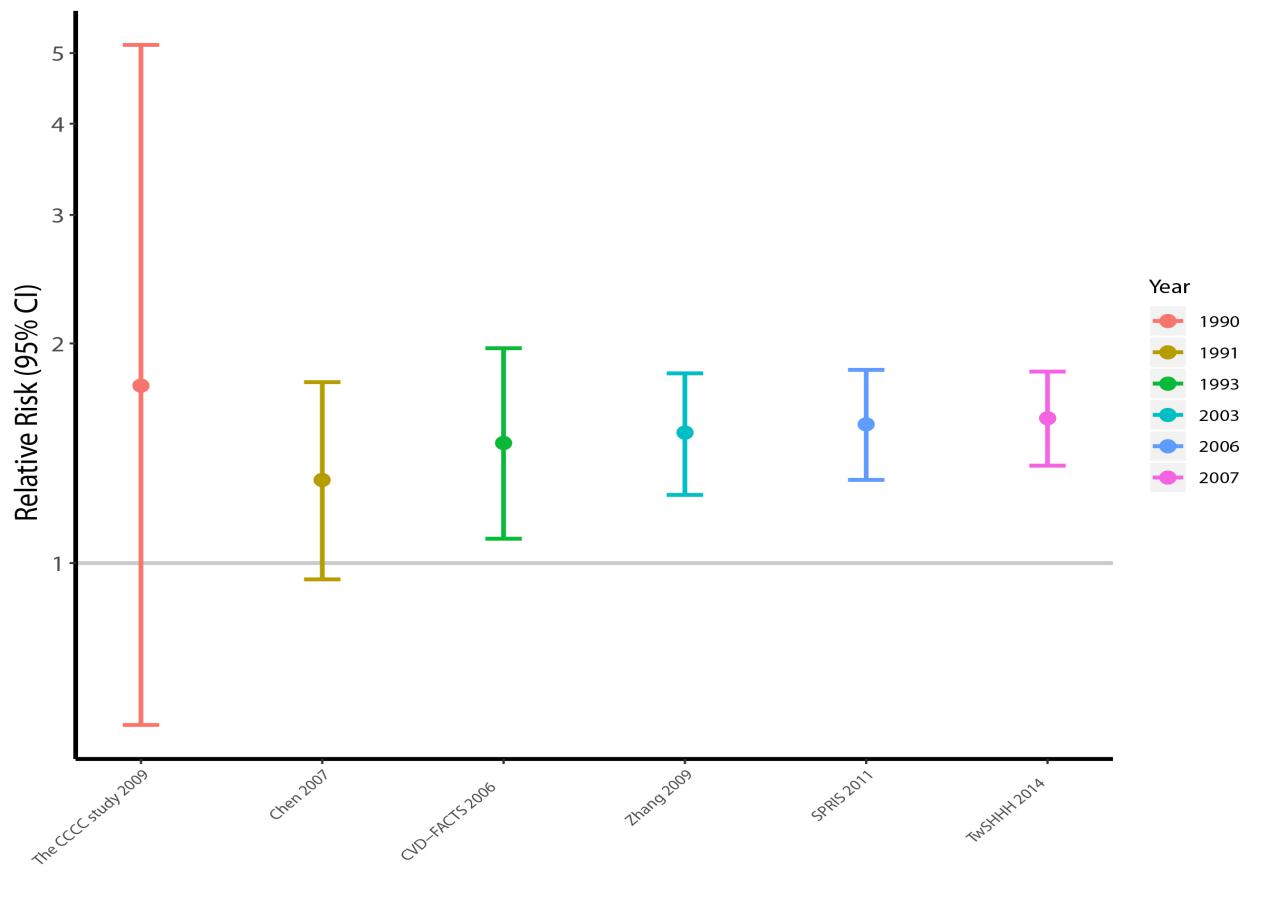


Figure S10. The role of cohort inclusion years for the association between metabolic syndrome and all stroke risk (*P*=0.376)


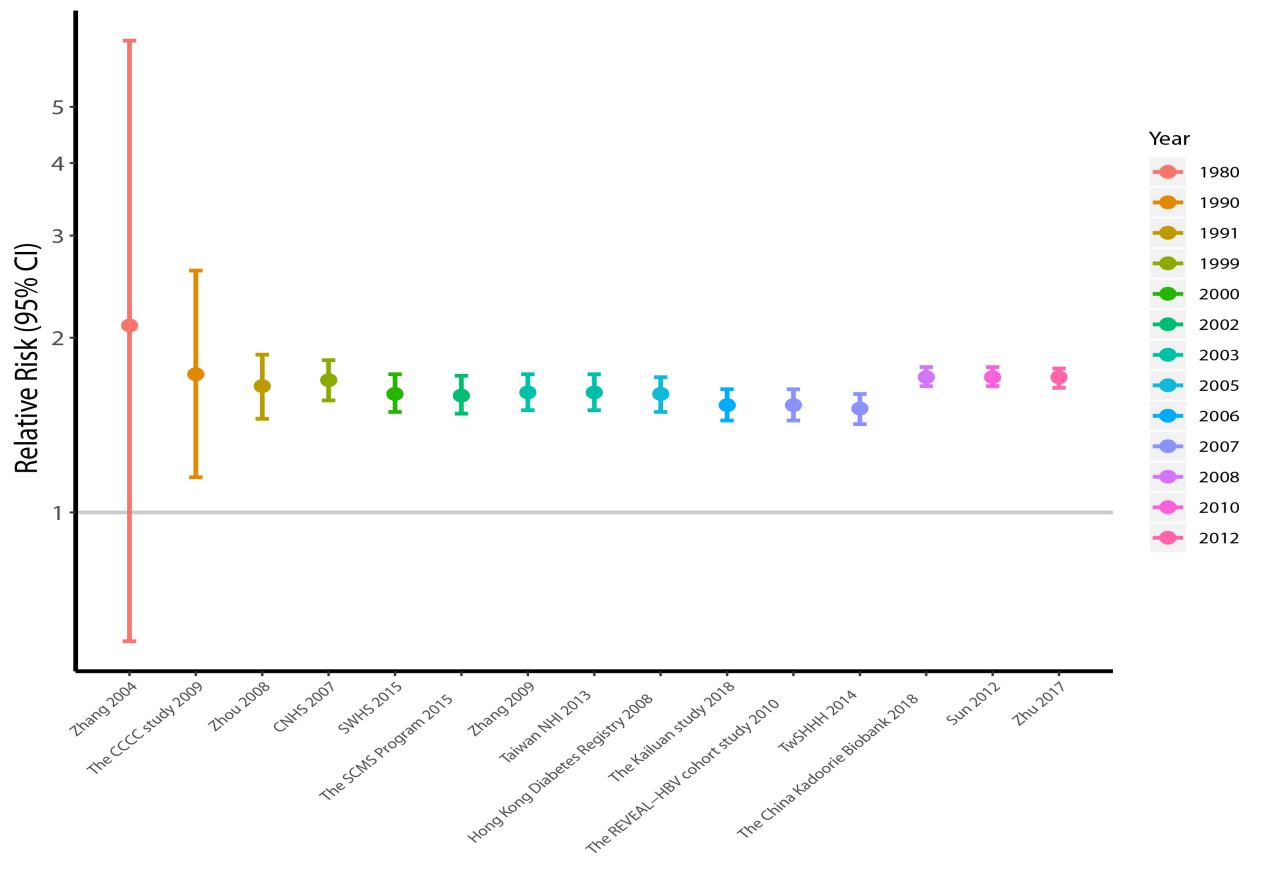


Figure S11. The role of cohort inclusion years for the association between obesity and all stroke risk (*P*=0.574)


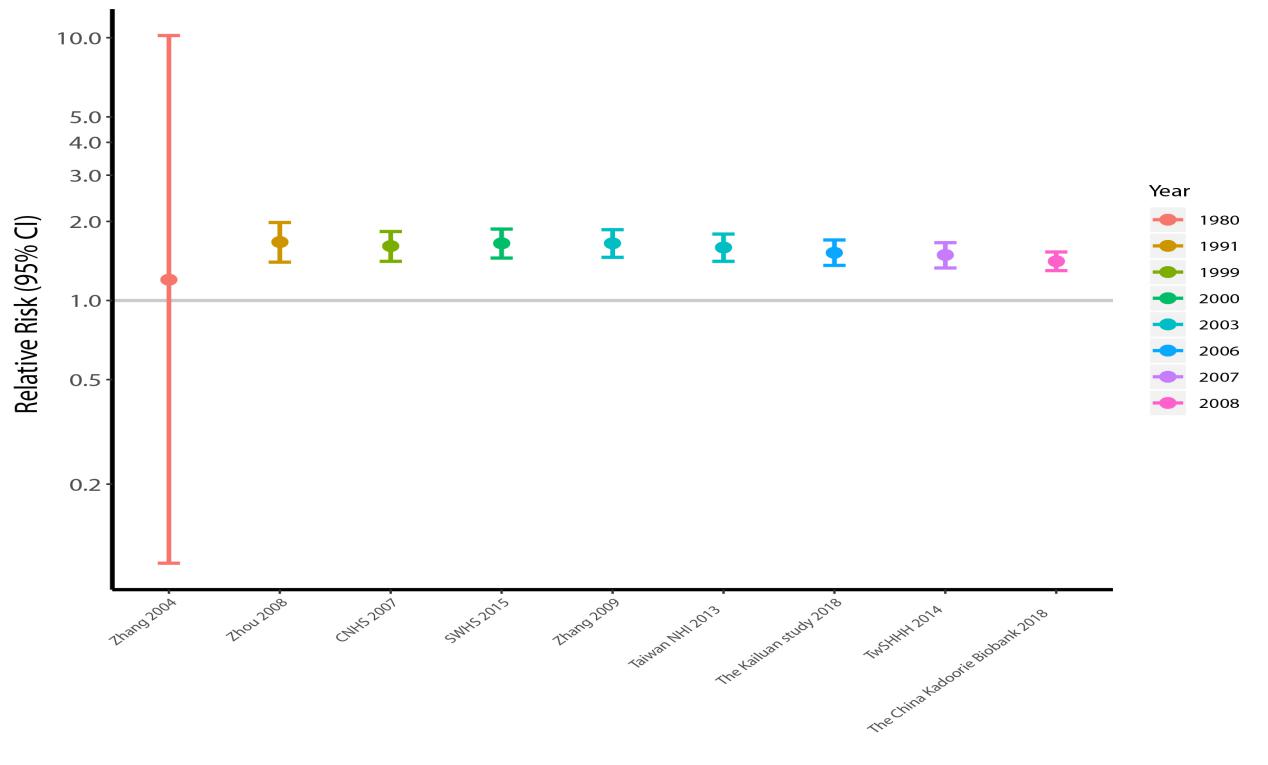


Figure S12. The role of cohort inclusion years for the association between obesity and HS risk (*P*=0.046)


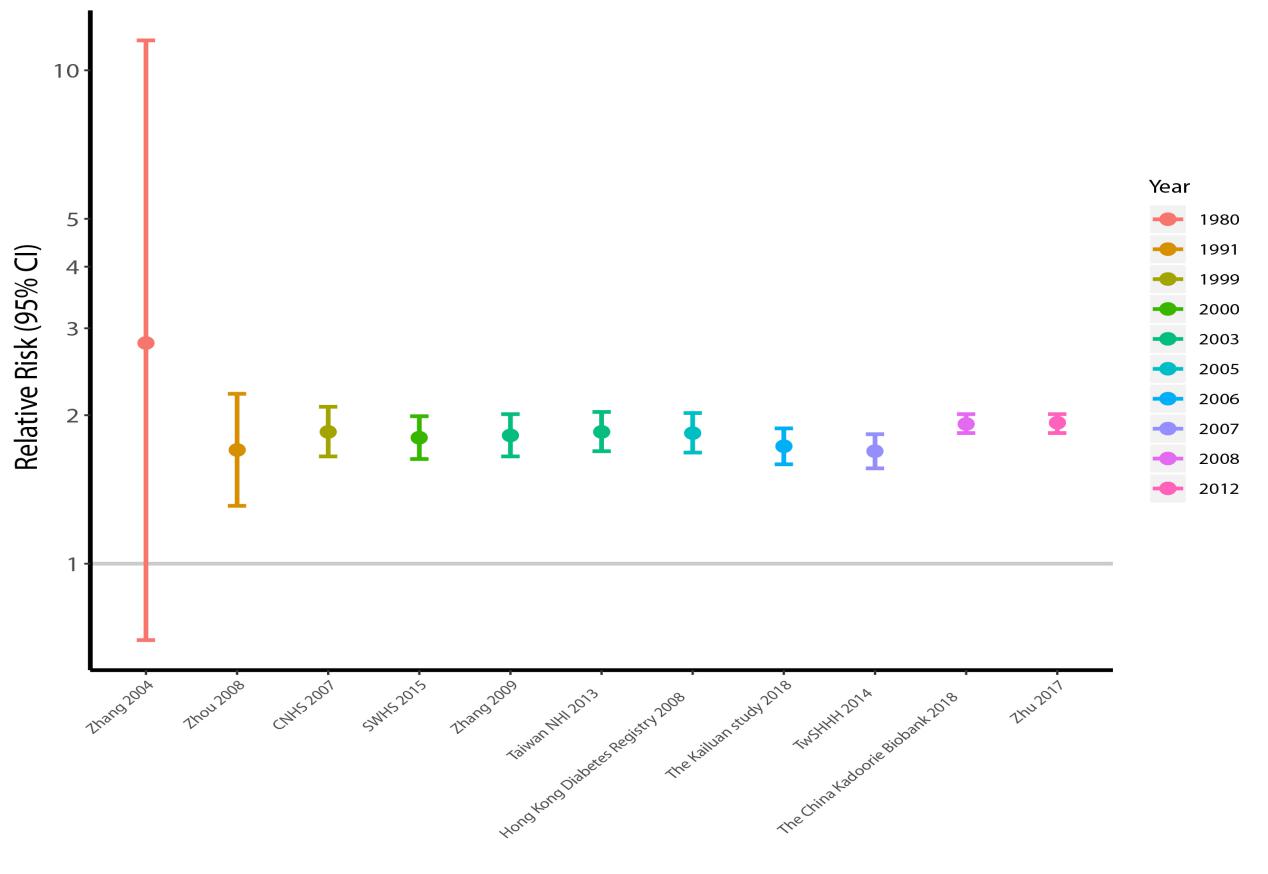


Figure S13. The role of cohort inclusion years for the association between obesity and IS risk (*P*=0.253)


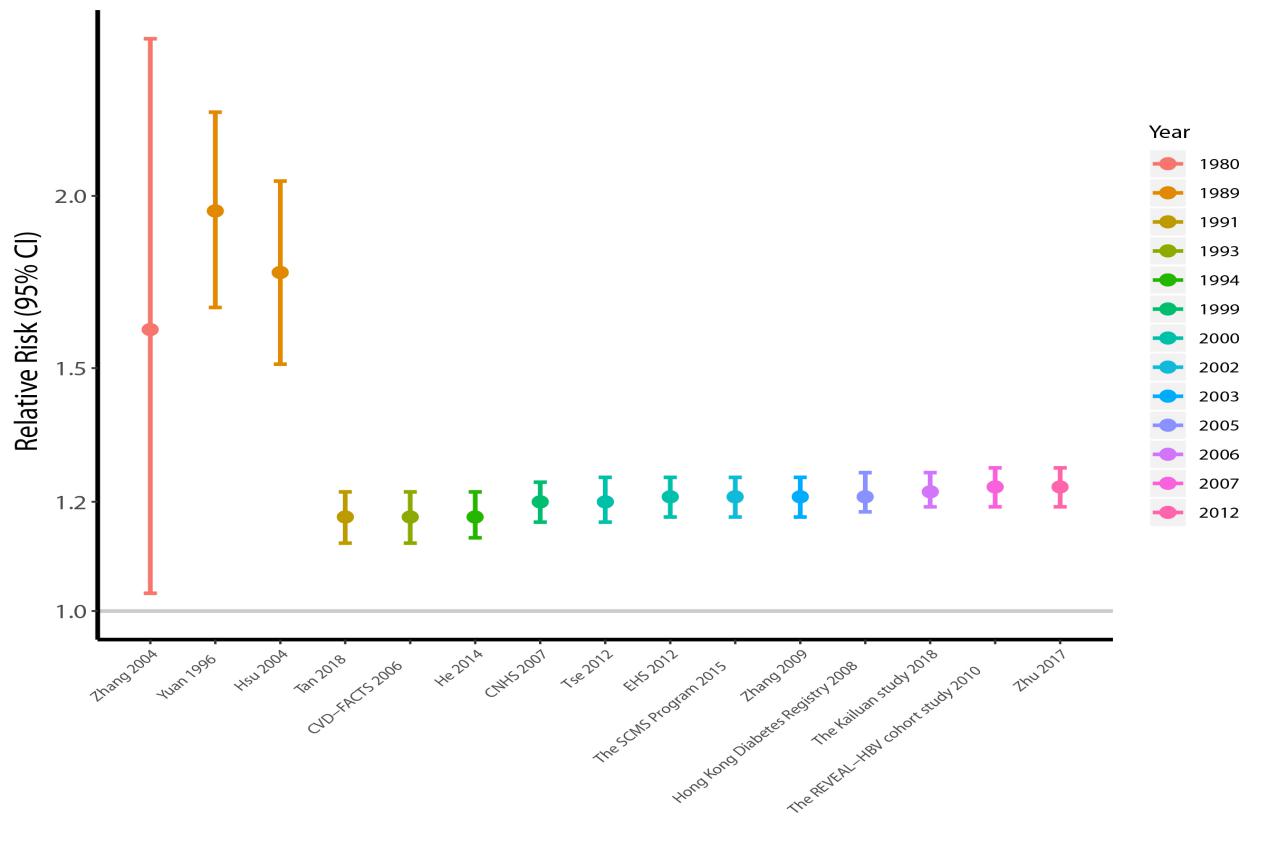


Figure S14. The role of cohort inclusion years for the association between smoker and all stroke risk (*P*=0.077)


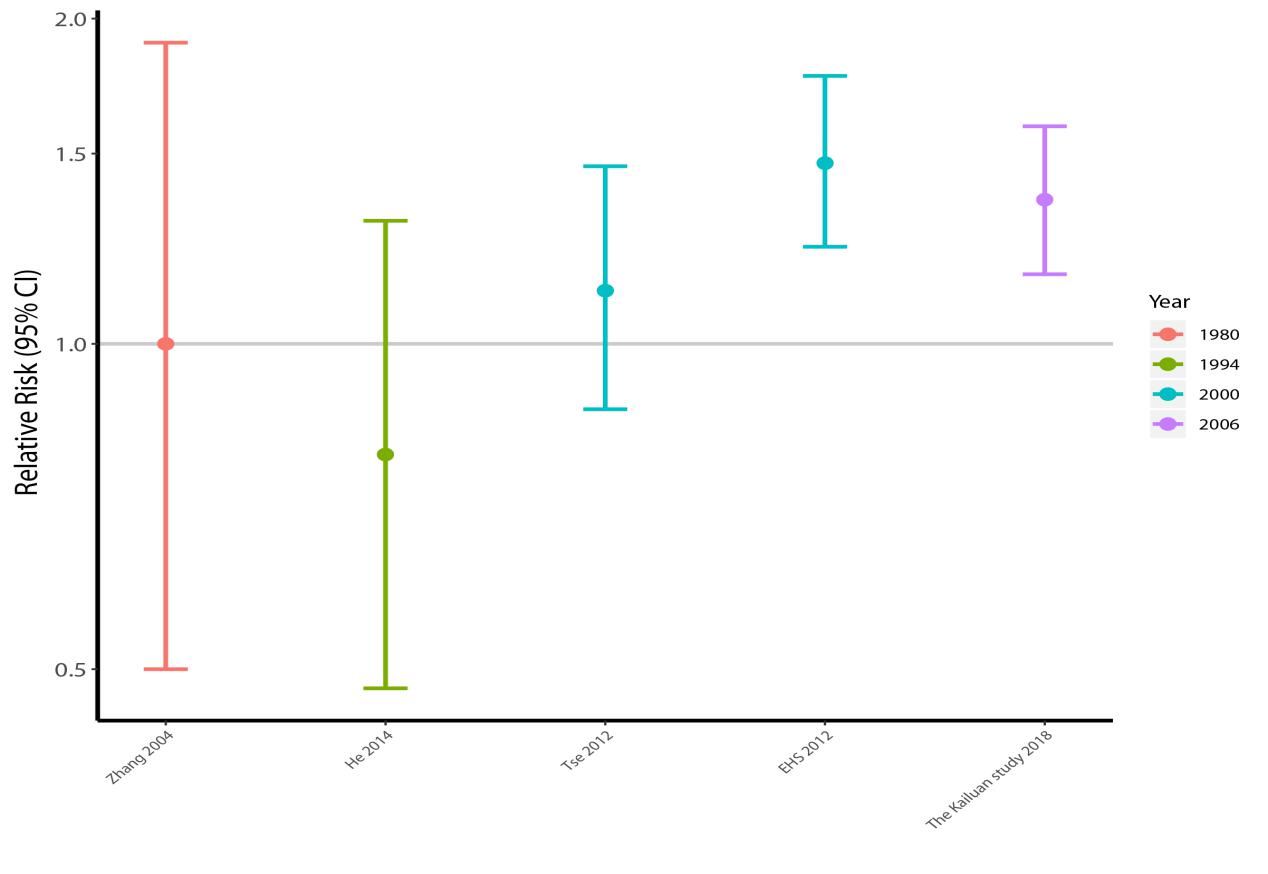


Figure S15. The role of cohort inclusion years for the association between smoker and HS risk (*P*=0.303)


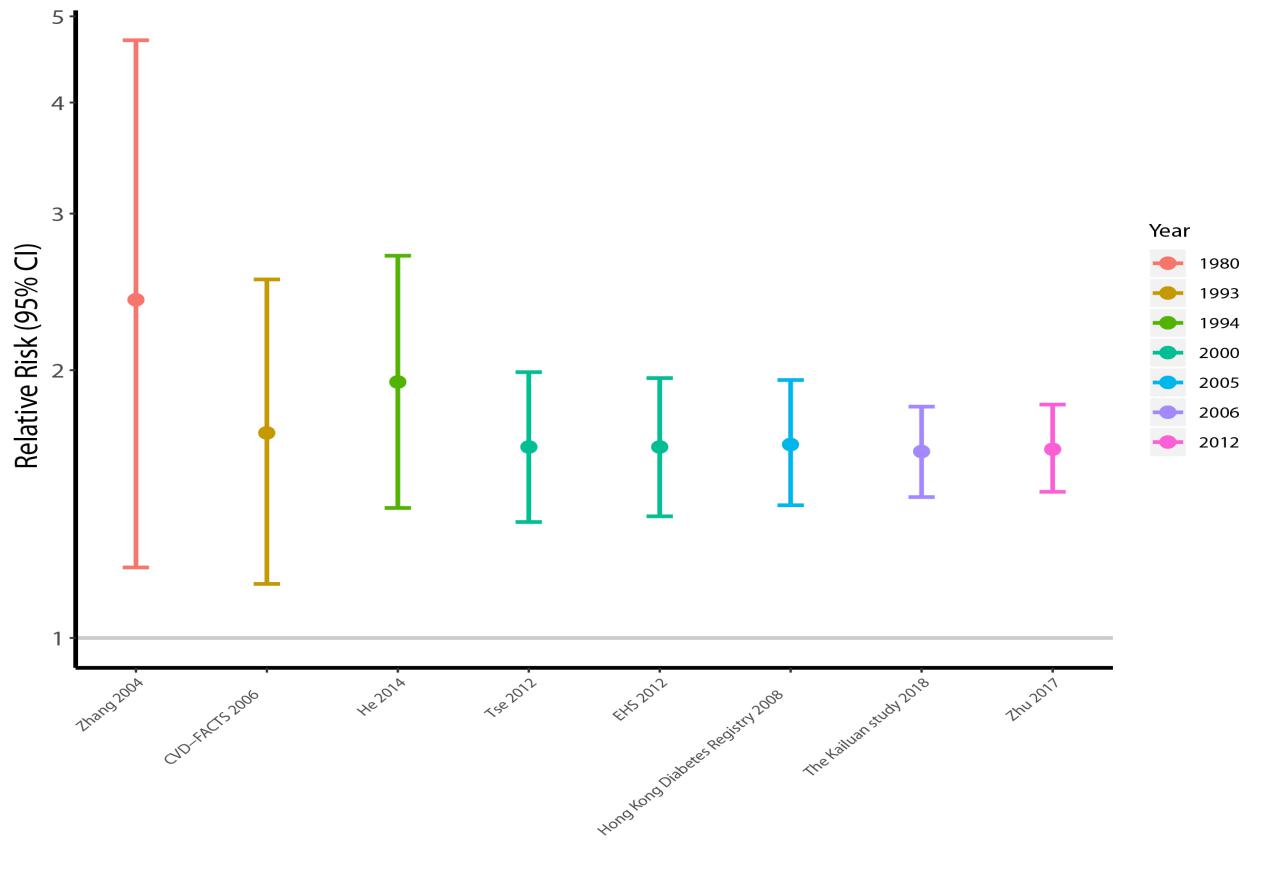


Figure S16. The role of cohort inclusion years for the association between smoker and IS risk (*P*=0.378)
