# Supplementary material for: Identification of Risk Factors for Stroke in China: A Meta-Analysis of Prospective Cohort Studies
Source: Front Neurol. 2022 Mar 18;13:847304. doi: 10.3389/fneur.2022.847304 (PMC8972128; doi:10.3389/fneur.2022.847304)
Supplement: Supplementary file 5 [file Table_5.DOCX]

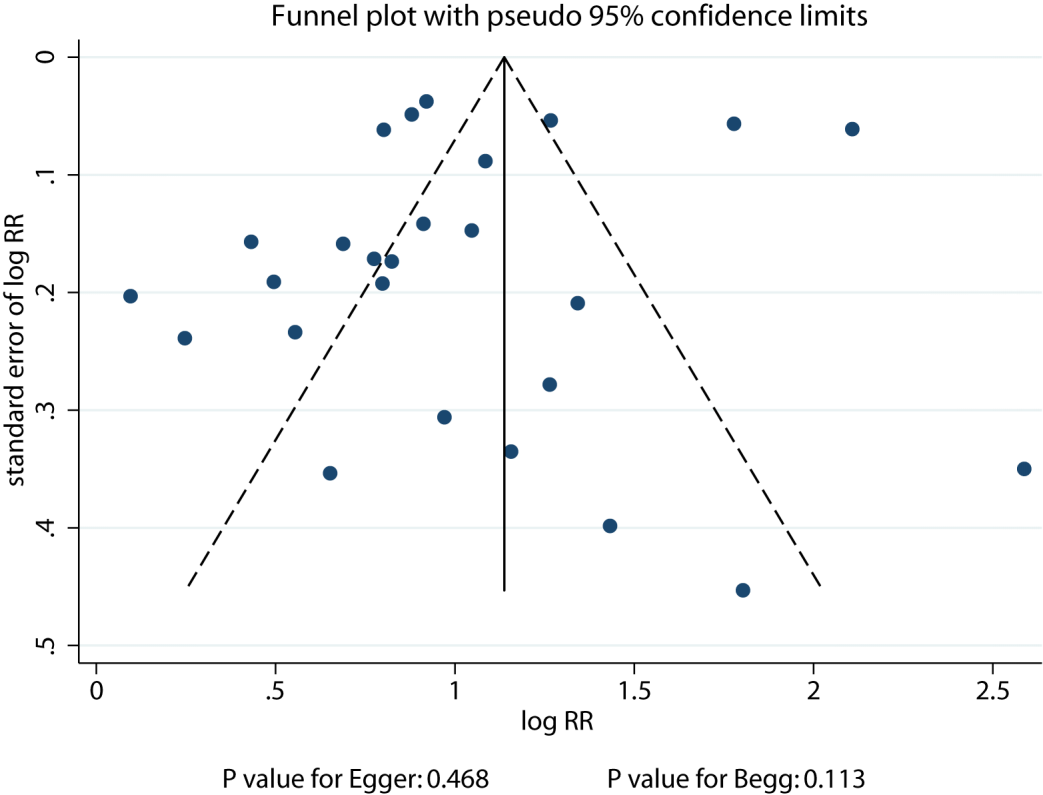


Figure S1. Funnel plot for hypertension


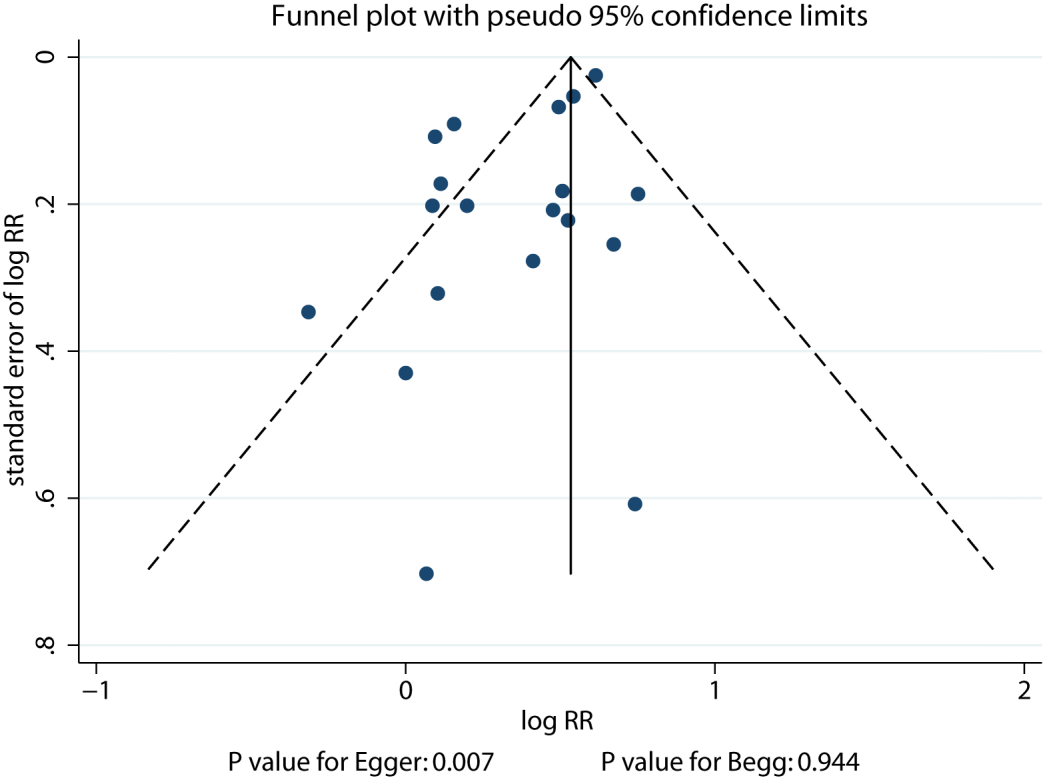


Figure S2. Funnel plot for obesity


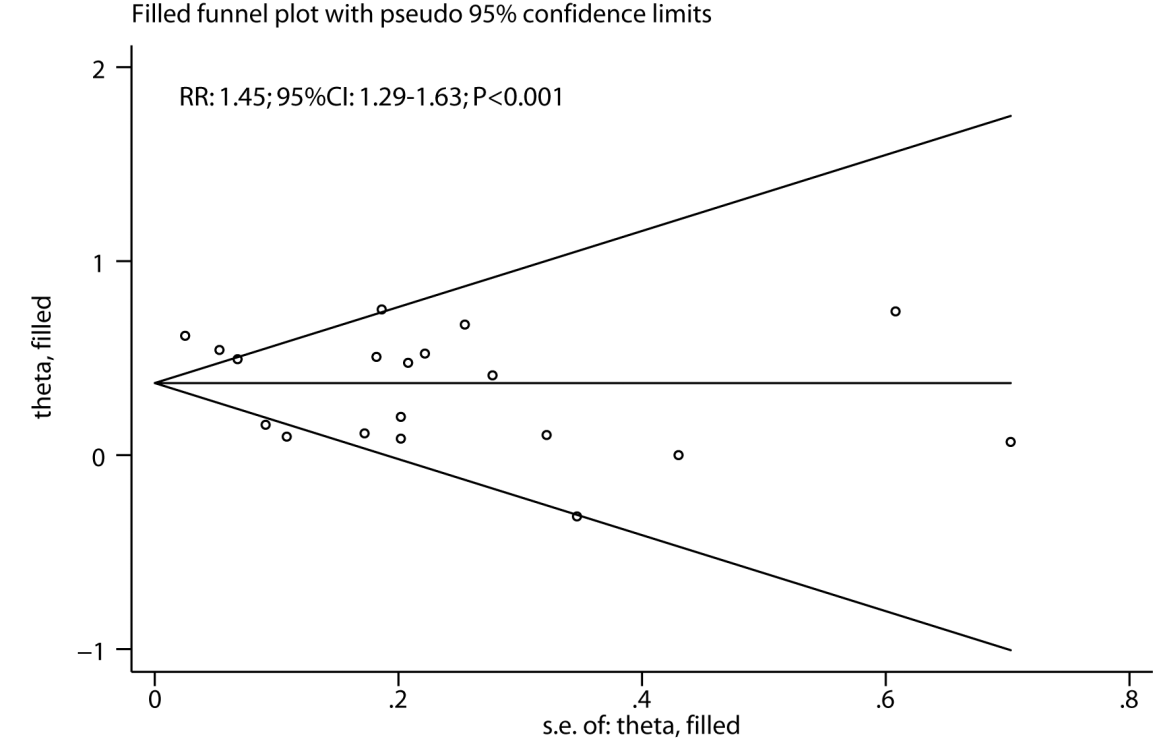


Figure S3. Trim and fill for obesity


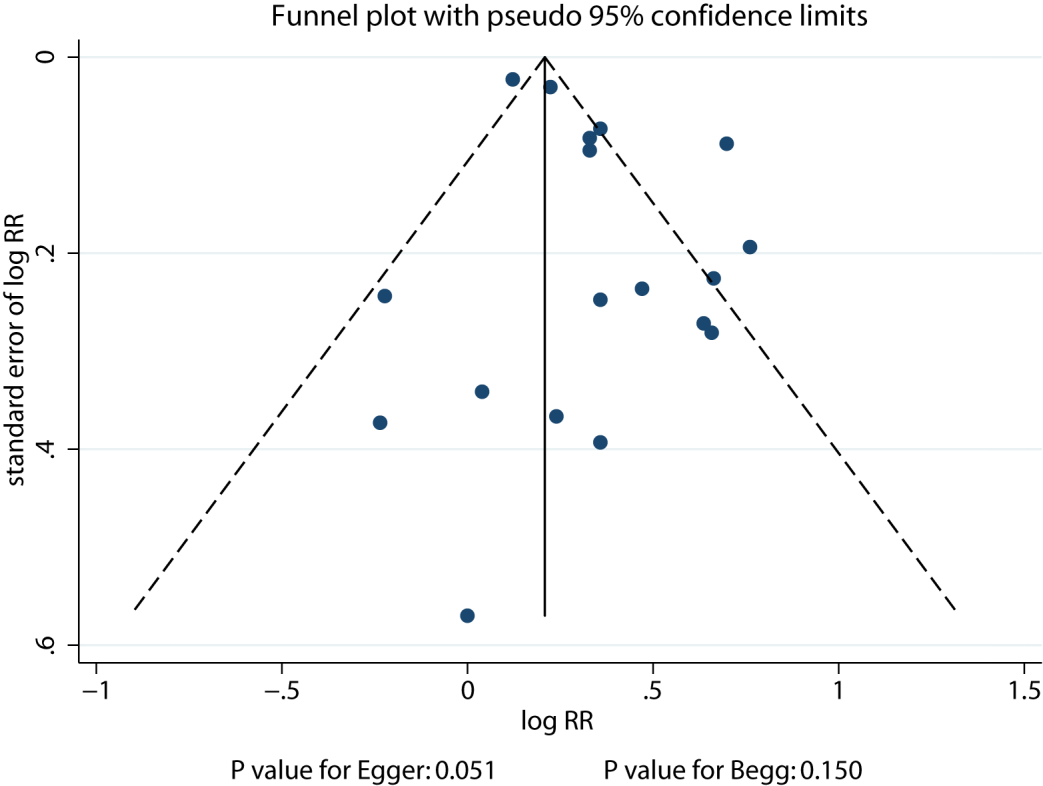


Figure S4. Funnel plot for smoking


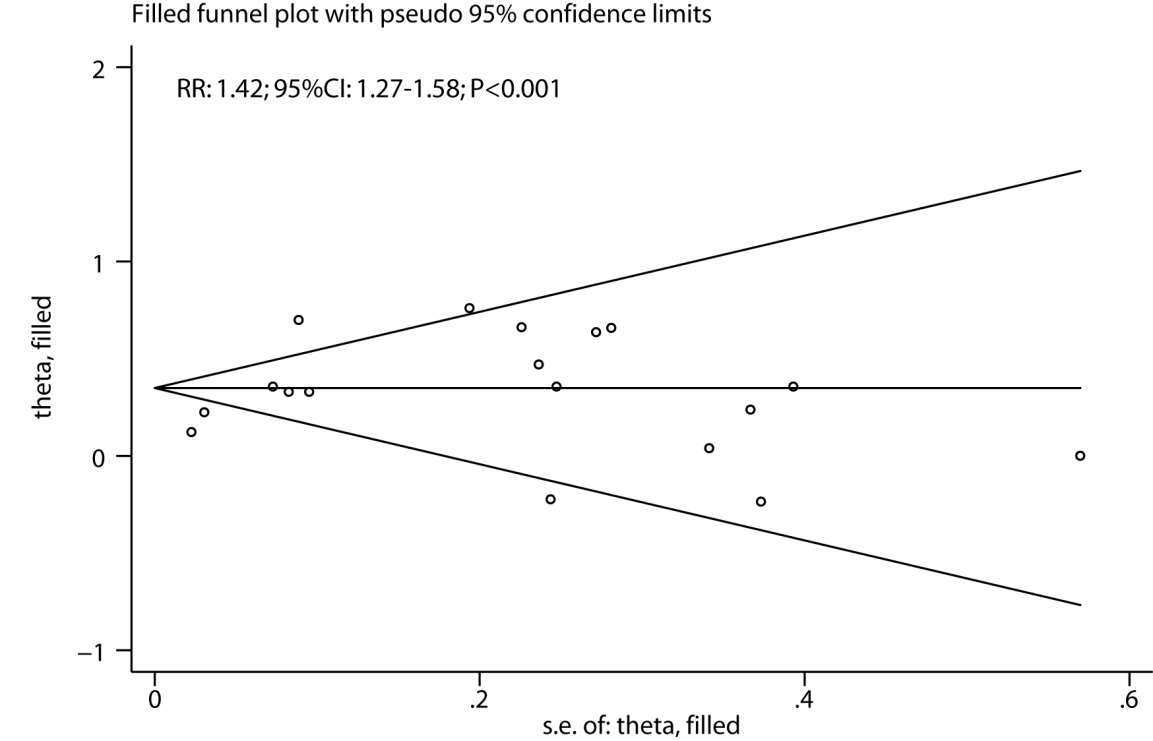


Figure S5. Trim and fill for smoking
