# Supplementary material for: Identification of Risk Factors for Stroke in China: A Meta-Analysis of Prospective Cohort Studies
Source: Front Neurol. 2022 Mar 18;13:847304. doi: 10.3389/fneur.2022.847304 (PMC8972128; doi:10.3389/fneur.2022.847304)
Supplement: Supplementary file 6 [file Table_6.DOCX]

Table S1. The results regarding the findings of this study and previous meta-analyses

| Variables | Our study | Previous systematic reviews |
| --- | --- | --- |
| Alcohol | All stroke: non-significant association; IS: protective effect;  HS: non-significant association | IS: light and moderate alcohol-protective effect, heavy alcohol-harmful effect; HS: light and moderate alcohol-non-significant associations, heavy alcohol-harmful effect [S1] |
| CKD | All stroke: harmful effect; IS: harmful effect; HS: harmful effect | Stroke risk increases linearly and additively with declining GFR and increasing albuminuria [S2] |
| DM | All stroke: harmful effect; IS: harmful effect; HS: non-significant association | DM was associated with greater risk of stroke in both men and women, and the risk of stroke in women was greater than in men [S3] |
| Elderly | All stroke: harmful effect | - |
| Gender (female vs male) | All stroke: non-significant association; IS: non-significant association; HS: female with less risk than male | - |
| Hyperglycemia | All stroke: harmful effect | Chronic hyperglycemia is associated with an increased risk for stroke [S4] |
| Hypertension | All stroke: harmful effect; IS: harmful effect; HS: harmful effect | All stroke: harmful effect; IS: harmful effect; HS: harmful effect [S5] |
| Metabolic Syndrome | All stroke: harmful effect; IS: harmful effect; HS: non-significant association | Metabolic syndrome might be an important risk factor of stroke, particularly among women and those with ischemic stroke [S6] |
| Obesity | All stroke: harmful effect; IS: harmful effect; HS: harmful effect | Both overweight and obesity increase the risk of stroke with a J-shaped dose-response relation, and the nadir of the curve was observed at BMI 23-24 kg/m^2^ [S7] |
| Sleep time (> 7.5 hours) | All stroke: harmful effect; IS: harmful effect; HS: non-significant association | The pooled RR was 1.05 (95% CI, 1.01-1.09) per 1-hour reduction and 1.18 (95% CI, 1.14-1.21) per 1-hour increment of sleep duration on the risk of stroke [S8] |
| Sleep time (< 6.5 hours) | All stroke: non-significant association; IS: non-significant association; HS: non-significant association |  |
| Smoking | All stroke: harmful effect; IS: harmful effect; HS: non-significant association | Stroke has a dose-dependent relationship with smoking, regardless of status as a current smoker or passive smoker [S9]. |
| Sleep behavior disorder | All stroke: harmful effect; IS: harmful effect; HS: harmful effect | - |
| TC | All stroke: non-significant association; IS: harmful effect; HS: non-significant association | IS: harmful effect for non-East Asians and non-significant association for East Asians; HS: harmful effect for non-East Asians and non-significant association for East Asians [S10] |
| TG | All stroke: harmful effect; IS: harmful effect; HS: non-significant association | All stroke: harmful effect [S11] |
| HDL | All stroke: non-significant association; IS: non-significant association; HS: non-significant association | All stroke: non-significant association [S12] |
| LDL | All stroke: non-significant association; IS: harmful effect; HS: non-significant association | All stroke: harmful effect [S11] |
| non-HDL | All stroke: harmful effect; IS: harmful effect; HS: non-significant association | - |
| TC/HDL | All stroke: harmful effect; IS: harmful effect; HS: non-significant association | - |
| LDL/HDL | All stroke: harmful effect; IS: harmful effect; HS: non-significant association | - |
| TG/HDL | All stroke: harmful effect; IS: harmful effect; HS: non-significant association | - |
| Lipoprotein | All stroke: harmful effect | All stroke: harmful effect [S13] |
| Apo-I | All stroke: non-significant association | - |
| ApoB | All stroke: non-significant association | - |
| Hypertriglyceridemia | All stroke: non-significant association; IS: non-significant association; HS: non-significant association | - |
| Family history of CVD | All stroke: non-significant association | - |
| Atrial fibrillation | All stroke: harmful effect | All stroke: harmful effect; IS: harmful effect [S14] |
| Total homocysteine | All stroke: non-significant association | IS: harmful effect; HS: harmful effect [S15] |
| CRP | All stroke: harmful effect; IS: harmful effect; HS: non-significant association | IS: harmful effect [S16] |
| CVD history | All stroke: harmful effect; IS: harmful effect; HS: harmful effect | - |
| Heart rate | All stroke: non-significant association; IS: non-significant association; HS: non-significant association | All stroke: harmful effect [S17] |
| Vegetable-Rich Diet | All stroke: non-significant association | All stroke: protective effect [S18] |
| Fruit-Rich Diet | All stroke: protective effect; IS: protective effect; HS: protective effect |  |
| Meat-Rich Diet | All stroke: non-significant association | The pooled relative risks (95% confidence intervals) for total, red, processed and white meat consumption and total stroke incidence were 1.18 (1.09-1.28), 1.11 (1.03-1.20), 1.17 (1.08-1.25), and 0.87 (0.78-0.97), respectively. Total meat consumption (0.97 [0.85-1.11]) and red meat consumption 0.87 (0.64-1.18) were not significantly associated with stroke-related death [S19] |
| Uric acid | All stroke: non-significant association; IS: non-significant association; HS: non-significant association | All stroke: harmful effect [S20] |

**Reference:**

S1. Larsson SC, Wallin A, Wolk A, et al. Differing association of alcohol consumption with different stroke types: a systematic review and meta-analysis. BMC Med 2016;14:178.

S2. Masson P, Webster AC, Hong M, et al. Chronic kidney disease and the risk of stroke: a systematic review and meta-analysis. Nephrol Dial Transplant. 2015;30:1162-9.

S3. Peters SA, Huxley RR, Woodward M. Diabetes as a risk factor for stroke in women compared with men: a systematic review and meta-analysis of 64 cohorts, including 775,385 individuals and 12,539 strokes. Lancet. 2014;383:1973-80.

S4. Zhang Y, Hu G, Yuan Z, et al. Glycosylated hemoglobin in relationship to cardiovascular outcomes and death in patients with type 2 diabetes: a systematic review and meta-analysis. PLoS One. 2012;7:e42551.

S5. He J, Klag MJ, Wu Z, et al. Stroke in the People's Republic of China. II. Meta-analysis of hypertension and risk of stroke. Stroke. 1995;26:2228-32

S6. Li X, Li X, Lin H, et al. Metabolic syndrome and stroke: A meta-analysis of prospective cohort studies. J Clin Neurosci. 2017;40:34-38.

S7. Liu X, Zhang D, Liu Y, et al. A J-shaped relation of BMI and stroke: Systematic review and dose-responsemeta-analysis of 4.43 million participants. Nutr Metab Cardiovasc Dis. 2018;28: 1092-1099.

S8. Yin J, Jin X, Shan Z, et al. Relationship of Sleep Duration With All-Cause Mortality and Cardiovascular Events: A Systematic Review and Dose-Response Meta-Analysis of Prospective Cohort Studies. J Am Heart Assoc. 2017 Sep 9;6(9). pii: e005947.

S9. Pan B, Jin X, Jun L, The relationship between smoking and stroke: A meta-analysis. Medicine (Baltimore). 2019;98:e14872.

S10. Xie L, Wu W, Chen J, et al. Cholesterol Levels and Hemorrhagic Stroke Risk in East Asian Versus Non-East Asian Populations: A Systematic Review and Meta-Analysis. Neurologist. 2017; 22:107-115.

S11. Labreuche J, Deplanque D, Touboul PJ, et al. Association between change in plasma triglyceride levels and risk of stroke and carotid atherosclerosis: systematic review and meta-regression analysis. Atherosclerosis. 2010;212:9-15.

S12. Huxley RR, Barzi F, Lam TH, et al. Isolated low levels of high-density lipoprotein cholesterol are associated with an increased risk of coronary heart disease: an individual participant data meta-analysis of 23 studies in the Asia-Pacific region. Circulation. 2011;124: 2056-64.

S13. Lp-PLA(2) Studies Collaboration, Thompson A, Gao P, Orfei L, et al. Lipoprotein-associated phospholipase A(2) and risk of coronary disease, stroke, and mortality: collaborative analysis of 32 prospective studies. Lancet. 2010;375:1536-44.

S14. Odutayo A, Wong CX, Hsiao AJ, et al. Atrial fibrillation and risks of cardiovascular disease, renal disease, and death: systematic review and meta-analysis. BMJ 2016;354:i4482.

S15. He Y, Li Y, Chen Y, et al. Homocysteine level and risk of different stroke types: a meta-analysis of prospective observational studies. Nutr Metab Cardiovasc Dis. 2014;24: 1158-65.

S16. Emerging Risk Factors Collaboration, Kaptoge S, Di Angelantonio E, Lowe G, et al. C-reactive protein concentration and risk of coronary heart disease, stroke, and mortality: an individual participant meta-analysis. Lancet. 2010;375:132-40.

S17. Aune D, Sen A, ó'Hartaigh B, et al. Resting heart rate and the risk of cardiovascular disease, total cancer, and all-cause mortality - A systematic review and dose-response meta-analysis of prospective studies. Nutr Metab Cardiovasc Dis. 2017;27:504-517.

S18. He FJ, Nowson CA, MacGregor GA. Fruit and vegetable consumption and stroke: meta-analysis of cohort studies. Lancet. 2006;367:320-6.

S19. Kim K, Hyeon J, Lee SA, et al. Role of Total, Red, Processed, and White Meat Consumption in StrokeIncidence and Mortality: A Systematic Review and Meta-Analysis of Prospective Cohort Studies. J Am Heart Assoc. 2017 Aug 30;6(9). pii: e005983.

S20. Zhong C, Zhong X, Xu T, et al. Sex-Specific Relationship Between Serum Uric Acid and Risk of Stroke: A Dose-Response Meta-Analysis of Prospective Studies. J Am Heart Assoc. 2017 Mar 29;6(4). pii: e005042.
